# Genome-Wide Maps of Mononucleosomes and Dinucleosomes Containing Hyperacetylated Histones of *Aspergillus fumigatus*

**DOI:** 10.1371/journal.pone.0009916

**Published:** 2010-03-26

**Authors:** Hiromi Nishida, Takayuki Motoyama, Yutaka Suzuki, Shogo Yamamoto, Hiroyuki Aburatani, Hiroyuki Osada

**Affiliations:** 1 Agricultural Bioinformatics Research Unit, Graduate School of Agricultural and Life Sciences, University of Tokyo, Tokyo, Japan; 2 Chemical Biology Core Facility, Advanced Science Institute, RIKEN, Wako, Saitama, Japan; 3 Department of Medical Genome Sciences, Graduate School of Frontier Sciences, University of Tokyo, Kashiwa, Japan; 4 Research Center for Advance Science and Technology, University of Tokyo, Tokyo, Japan; Texas A&M University, United States of America

## Abstract

It is suggested that histone modifications and/or histone variants influence the nucleosomal DNA length. We sequenced both ends of mononucleosomal and dinucleosomal DNA fragments of the filamentous fungus *Aspergillus fumigatus*, after treatment with the histone deacetylase inhibitor trichostatin A (TSA). After mapping the DNA fragments to the genome, we identified >7 million mononucleosome positions and >7 million dinucleosome positions. We showed that the distributions of the lengths of the mononucleosomal DNA fragments after 15-min and 30-min treatments with micrococcal nuclease (MNase) showed a single peak at 168 nt and 160 nt, respectively. The distributions of the lengths of the dinucleosomal DNA fragments after 15-min- and 30-min-treatment with MNase showed a single peak at 321 nt and 306 nt, respectively. The nucleosomal DNA fragments obtained from the TSA-treated cells were significantly longer than those obtained from the untreated cells. On the other hand, most of the genes did not undergo any change after treatment. Between the TSA-treated and untreated cells, only 77 genes had ≥2-fold change in expression levels. In addition, our results showed that the locations where mononucleosomes were frequently detected were conserved between the TSA-treated cells and untreated cells in the gene promoters (lower density of the nucleosomes). However, these locations were less conserved in the bodies (higher density of the nucleosomes) of genes with ≥2-fold changes. Our findings indicate that TSA influences the nucleosome positions, especially of the regions with high density of the nucleosomes by elongation of the nucleosomal DNA. However, most of the nucleosome positions are conserved in the gene promoters, even after treatment with TSA, because of the low density of nucleosomes in the gene promoters.

## Introduction

Eukaryotic genomic DNA is packaged with histone proteins to form chromatin [1], the most fundamental repeating unit of which is the nucleosome [2]. Nucleosomes consist of an octamer of histones, around which the DNA is wrapped in 1.65 turns [3]. Neighboring nucleosomes are separated by unwrapped linker DNA. The precise organization of this chromatin is of utmost importance for the maintenance of eukaryotic genomic DNA.

Generally, nucleosomal histone proteins are post-translationally modified [4]. Reversible histone acetylation, which is regulated by histone acetyltransferase [5] and deacetylase [6], is one such modification. The acetylation and deacetylation of the core histone tails plays an important role in the regulation of transcription [7], [8]. Trichostatin A (TSA) is a histone deacetylase inhibitor, which induces hyperacetylation of histone proteins [9], [10].

Analyses using high-density genomic tiling arrays or massively parallel DNA sequencers have led to the high-resolution mapping of genome-wide nucleosome positions [11]–[22]. Genome-wide nucleosome maps have revealed the relationship between nucleosome density (particularly the presence or absence of nucleosomes in gene promoters) and gene expression. In our previous study [16], 7,715,001 mononucleosomal and 8,565,279 dinucleosomal DNA fragments of the fungus *Aspergillus fumigatus* were mapped using a massively parallel DNA sequencer.

Our previous analyses indicated that the distribution of the mononucleosomal DNA fragment lengths showed 2 peaks at 135 nt and at 150 nt [16]. Although the 2 peaks were found in the gene bodies of active and inactive genes and the inactive gene promoters, the peak at 150 nt was not found in the active gene promoters [16], suggesting that the nucleosomal DNA length of the active gene promoters is related to gene expression. In addition, the distribution of the dinucleosomal DNA fragment lengths showed a single peak at 285 nt [16]. Considering the ratio of the mononucleosome mapping number to the dinucleosome mapping number, the sensitivity to micrococcal nuclease (MNase) was estimated [23]. Based on this estimation, the sensitivity of the active promoters to MNase is not likely to be the sole reason for the loss of the farthest peak [23]. The ratio can be useful for detection of MNase effect.

In the present study, mononucleosomal and dinucleosomal DNA fragments of the TSA-treated cells of *A. fumigatus* were mapped to its genome, and they were compared to those of the untreated cells.

## Results

### Distribution of mononucleosomal and dinucleosomal DNA fragment lengths

**Figure 1:**
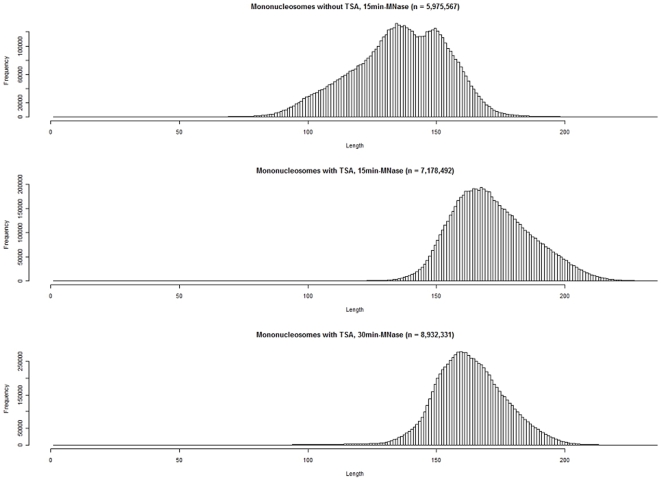
Histograms of the mononucleosomal DNA fragment lengths from trichostatin A (TSA)-treated cells of *Aspergillus fumigatus*. Top, mononucleosomal DNA fragments from untreated cells from our previous study [16]; middle, mononucleosomal DNA fragments from TSA-treated cells (15-min treatment with MNase); bottom, mononucleosomal DNA fragments from TSA-treated cells (30-min treatment with MNase). The distribution of the DNA fragment lengths from the normal cells showed 2 peaks (135 nt and 150 nt). The distribution of the DNA fragment lengths from 15-min and the 30-min treatment with MNase had single peaks at 168 nt and 160 nt, respectively.

**Figure 2:**
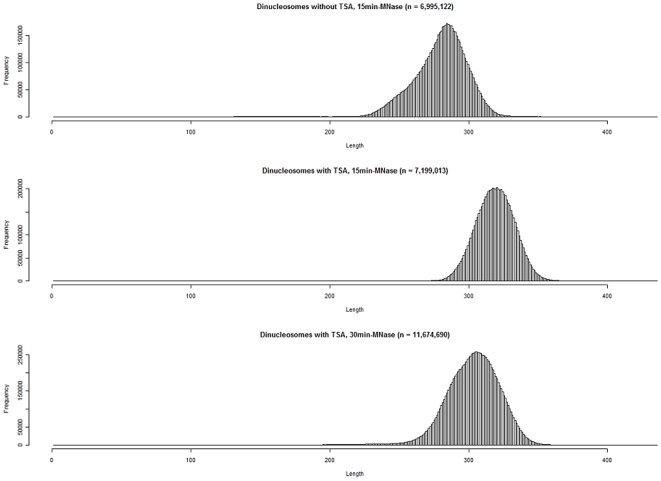
Histograms of the dinucleosomal DNA fragment lengths of the TSA-treated cells of *Aspergillus fumigatus*. Top, dinucleosomal DNA fragments from untreated cells in our previous study [16]; middle, dinucleosomal fragments from trichostatin A (TSA)-treated cells (15-min treatment with MNase); bottom, dinucleosomal fragments from TSA-treated cells (30-min treatment with MNase). The distribution of the DNA fragment lengths from the untreated cells showed a single peak at 285 nt. The distribution of the DNA fragment lengths from cells treated with TSA for 15 min and from the cells treated for 30 min had singles peak at 321 nt and 306 nt, respectively.

From the mapped data, we excluded the mononucleosomal and dinucleosomal DNA fragments that were longer than 236 nt and 436 nt, respectively Accordingly, we identified 9,487,154 and 11,086,845 mononucleosomal DNA fragments and 9,197,430 and 14,382,898 dinucleosomal DNA fragments, after 15-min and 30-min treatment with MNase, respectively. After excluding the completely overlapping DNA fragments, we identified 7,178,492 and 8,932,331 mononucleosome positions and 7,199,013 and 11,674,690 dinucleosome positions, after 15-min and 30-min treatment with MNase, respectively. The distribution of mononucleosomal DNA fragment lengths had a single peak at 168 nt and 160 nt, after 15-min and 30-min-treatment with MNase, respectively (Fig. 1). The distribution of dinucleosomal DNA fragment lengths had a single peak at 321 nt and 306 nt, after 15-min and 30-min treatment with MNase, respectively (Fig. 2).

### Identification of transcription start sites

The 7,496,630 DNA sequences (5′-end 34 bases) of the untreated cells were uniquely mapped to the *A. fumigatus* genome. Among the 7,496,630 sequence tags, we identified 6,968,134 that were identical to the genome sequences (34 bases matched perfectly). In this study, the 6,968,134 sequence tags were used to identify the transcription start sites (TSSs). We identified 372,259 different TSSs in the whole genome. Of these, 5,386 TSSs had >100 and 557 had >1000 sequence tags.

To compare the conservation levels of the mononucleosome positions around the TSSs, we calculated the squares of Pearson's correlation coefficients between the profiles of the mononucleosome mapping numbers of the TSA-treated cells (15-min treatment with MNase) and the untreated cells at 300 nt downstream and upstream of the 557 TSSs (with more than 1000 sequence tags). The results are shown in Fig. S1.

### Estimation of sensitivity to MNase

We identified 50 highly expressed (active) genes (Table S1) and 50 lowly expressed or silent (inactive) genes (Table S2) on the basis of the microarray data of the RNAs of the TSA-treated cells of *A. fumigatus*. The mononucleosome and dinucleosome mapping numbers and their ratios were calculated for each nucleotide position in the region from 1 kb upstream of the TSS to the translational end site for each of the 50 transcriptionally active and 50 inactive genes. The ratio of the mononucleosome mapping site number to the dinucleosome mapping site number was used as a marker for the sensitivity of nucleosomes to MNase [23]. The region 1 kb upstream from the TSS was designated as the gene promoter and the region downstream from the translational start to the end was designated as the gene body.

**Figure 3:**
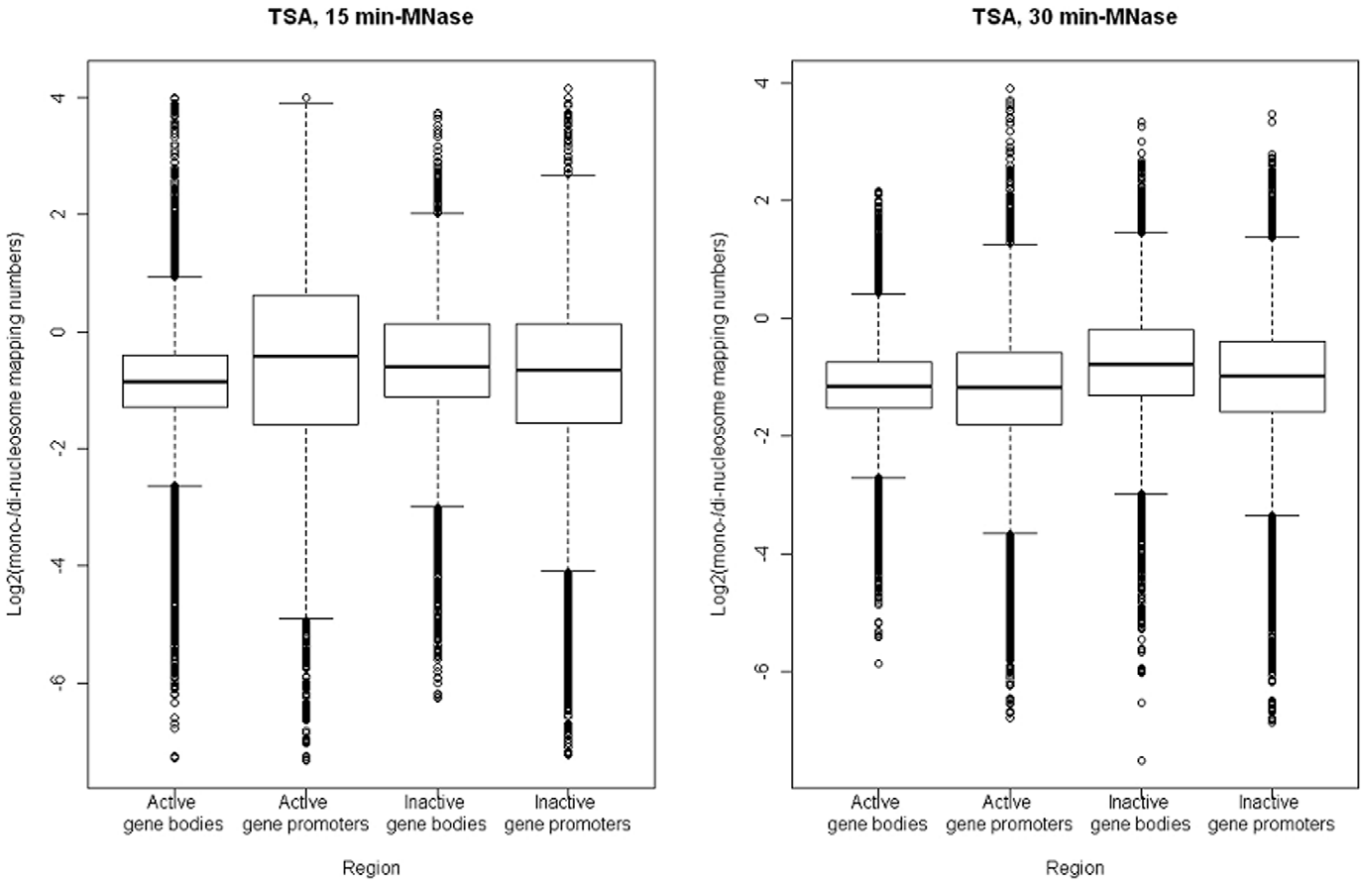
Boxplots of the ratios (log_2_ scale) of the mononucleosome mapping numbers to the dinucleosome mapping numbers for trichostatin A-treated cells of *Aspergillus fumigatus* in transcriptionally active and inactive gene bodies and promoters. The boxes are composed of the medians with the first and third quartiles. The dots indicate outliers.

The genomic nucleotide positions that exhibited a ratio of the mononucleosome mapping number to the dinucleosome mapping number, with both numbers being ≥1 for each nucleotide position in the active gene bodies, active gene promoters, inactive gene bodies, and inactive gene promoters were 80,381, 41,062, 47,933, and 40,421, respectively (15-min treatment with MNase), and 81,486, 47,960, 50,008, and 46,338, respectively (30-min-treatment with MNase). The median value of the ratios (log_2_ scale) in the active gene bodies, active gene promoters, inactive gene bodies, and inactive gene promoters was −0.86, −0.41, −0.59, and −0.66, respectively (15-min treatment with MNase and −1.16, −1.17, −0.79, and −0.98, respectively (30-min treatment with MNase (Fig. 3).

MNase sensitivity of the active gene promoters was the highest in the TSA-treated cells subjected to a 15-min treatment with MNase (Fig. 3). However, in the TSA-treated cells subjected to a 30-min treatment with MNase, the MNase sensitivity of the active promoters was similar to that of the active gene bodies and was lower than those of the inactive gene bodies and promoters (Fig. 3). Generally, the promoter region is more sensitive to nucleases than the gene body regions. In fact, the MNase sensitivity of the active gene promoters of untreated *A. fumigatus* cells was higher than that of the active gene bodies [16]. Thus, in this analysis, we compared the nucleosome map of the TSA-treated cells to that of the untreated cells, after subjecting them to a 15-min treatment with MNase.

### Comparison of conservation levels of mononucleosome positions

After comparison of gene expression levels between the TSA-treated and untreated cells of *A. fumigatus*, we selected 50 genes with constant expression, 49 with down-regulated expression (≥2-fold change), and 28 genes with up-regulated expression (≥2-fold change) (Tables S3 and S4). The mapping mononucleosome numbers and the TSSs of the 50 constantly expressed genes, the 49 down-regulated genes, and the 28 up-regulated genes are shown in Figs. S2, S3, and S4, respectively. As for each gene, the gene promoter (region of 1 kb upstream from the TSS) and the gene body (region from the translational start to the end) were considered.

**Figure 4:**
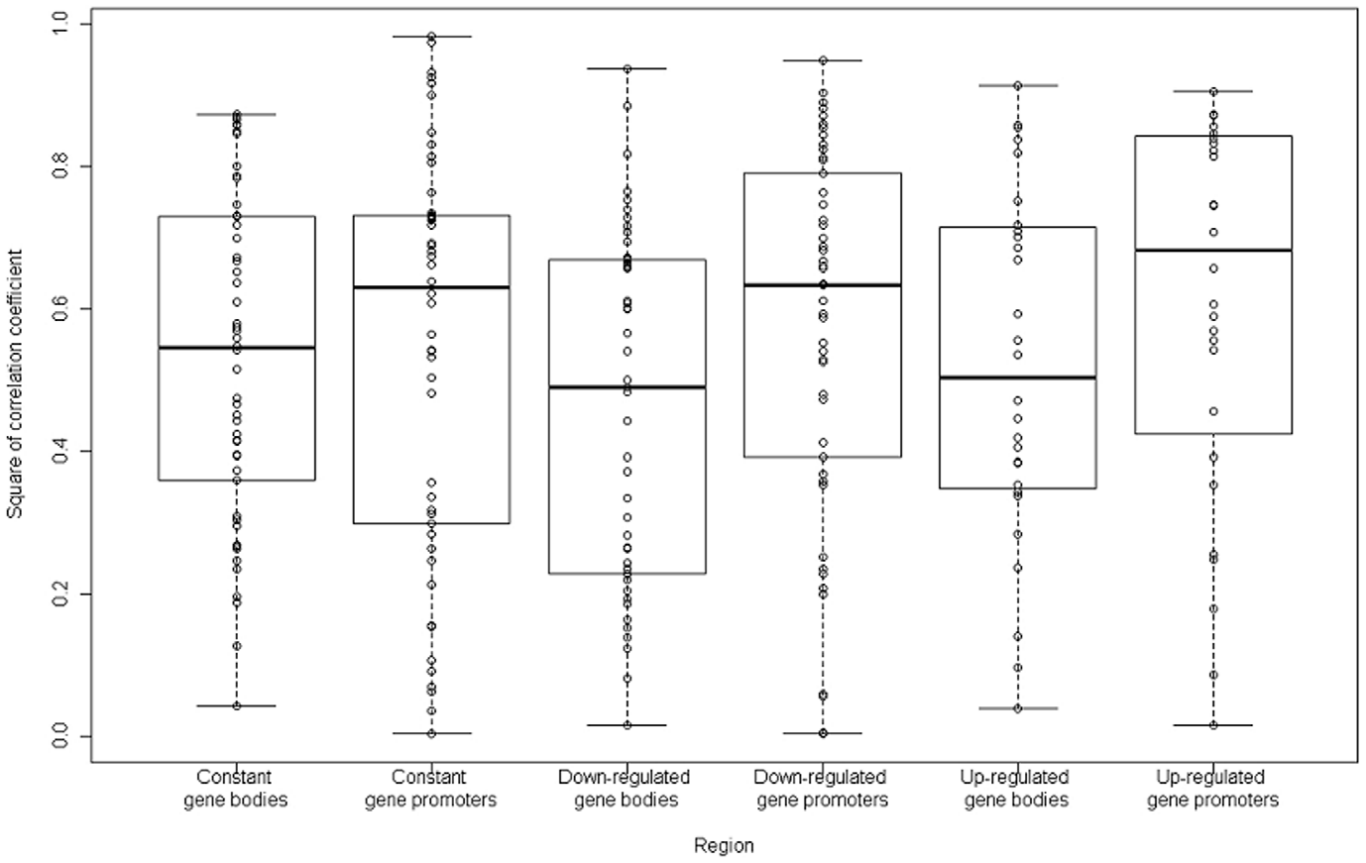
Boxplots of the squares of Pearson's correlation coefficient between the profiles of the trichostatin A-treated (15-min treatment with MNase) and untreated mononucleosome mapping numbers in the constantly expressed gene bodies, constantly expressed gene promoters, down-regulated gene bodies, down-regulated gene promoters, up-regulated gene bodies, and up-regulated gene promoters. The boxes are composed of the medians with the first and third quartiles. The dots indicate each of the squares of Pearson's correlation coefficient.

The medians of the squares of Pearson's correlation coefficients between the profiles of the mononucleosome mapping numbers of the TSA-treated cells (15-min treatment with MNase) and the untreated cells were 0.54, 0.63, 0.49, 0.63, 0.50, and 0.68 for the constantly expressed gene bodies, constantly expressed gene promoters, down-regulated gene bodies, down-regulated gene promoters, up-regulated gene bodies, and up-regulated gene promoters, respectively (Fig. 4). We performed Wilcoxon signed-rank test between the gene bodies and promoters. As the results, the *p*-values were 0.89, 0.034, and 0.21 in the constantly expressed genes, the down-regulated genes, and the up-regulated genes, respectively.

### Comparison of the expression levels of histones-related genes

**Figure 5:**
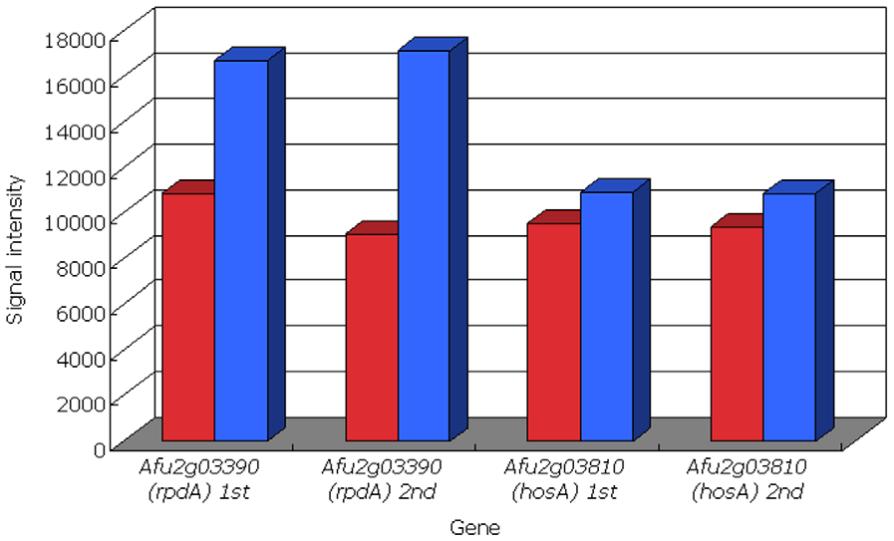
Transcription expression levels of the 2 histone deacetylase genes *rpdA* and *hosA* of *Aspergillus fumigatus*. Red and blue indicate the untreated and trichostatin A-treated cells, respectively. “1st” and “2nd” means each result of two technical replicates in the microarray analyses.

**Figure 6:**
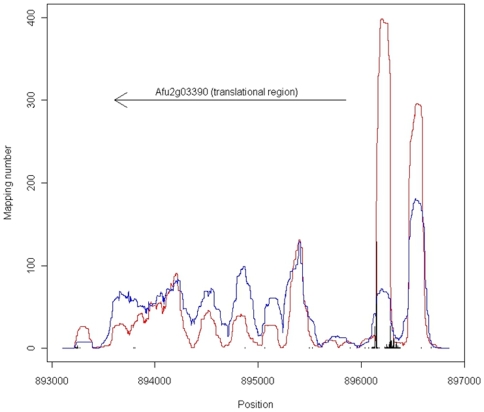
Mapping numbers of the mononucleosomes and transcription start sites within the histone deacetylase gene *rpdA* of *Aspergillus fumigatus*. Red and blue indicate the mononucleosome mapping numbers of the untreated and trichostatin A-treated cells, respectively. The arrow indicates the region from the translational start to the end. The bars indicate the transcription start sites and the mapping numbers. This gene locates on the chromosome 2 of *A. fumigatus*.

*Aspergillus fumigatus* has 31 histone-related genes (including 10 histone deacetylase-related genes) (Table S5). Among the 31 genes, the fold-change level of *rpdA* (*Afu2g03390*), a histone deacetylase gene, was the highest based on our microarray data (Table S5, Fig. 5). We compared the mapping numbers of the mononucleosomes and the TSSs of *Afu2g03390* between in the TSA-treated and untreated cells of *A. fumigatus*. In the untreated cells, the gene promoters had mononucleosomes whose locations were strictly fixed (Fig. 6). Around the fixed mononuclesomes, there was 1 major TSS and other minor sites (Fig. 6). On the other hand, in the TSA-treated cells, the fixed mononucleosome mapping numbers at the promoters decreased significantly and the locations were slightly variable (Fig. 6).

## Discussion

Graessle et al. [24] showed that the treatment of cells with TSA stimulates expression of the histone deacetylase coding gene *rpdA* (homolog of *RPD3* from *Saccharomyces cerevisiae*) of *Aspergillus nidulans*; however, TSA had no effect on the expression of *hosA* (homolog of *HOS2* from *Saccharomyces cerevisiae*), a gene that codes for histone deacetylase. The evolutionary conservation levels of *rpdA* and *hosA* are the second and third highest among the 110 proteins related to fungal histone-modifying protein complexes, respectively [25]. On the basis of our microarray data, we compared the expression levels of the homologous genes (*Afu2g03390* and *Afu2g03810*) of *rpdA* and *hosA* between the TSA-treated and untreated cells of *A. fumigatus* (Fig. 5). The fold-change level of *rpdA* (*Afu2g03390*) was the highest among the 31 histone-related genes (Table S5). The results of our microarray analysis (Fig. 5) were consistent with the results of the expression analysis of *A. nidulans* performed by Graessle et al. [24], indicating that TSA-treatment was also effective for the *A. fumigatus* cells used in our experiments. The *rpdA* is an essential gene for growth and development of *A. nidulans* [26]. It suggests that the function of RpdA is not completely inhibited in the TSA-treated cells of *Aspergillus*.

We checked the mapping numbers of the mononucleosomes and the TSSs of *Afu2g03390*. In the untreated cells of *A. fumigatus*, the gene promoters had mononucleosomes whose locations were strictly fixed (Fig. 6). In addition, there was 1 major TSS and other minor sites, which were located around the fixed mononucleosomes (Fig. 6). However, in the TSA-treated cells, the fixed mononucleosome mapping numbers at the promoters decreased significantly and the locations were slightly variable. The histone deacetylase inhibitor TSA induces hyperacetylation of histone proteins, which may result in the hyperacetylated histones being released from the gene promoters. These results strongly suggest that the histone acetylation level of nucleosomes in the gene promoter of *Afu2g03390* is directly related to gene regulation.

Our previous study indicated that the distribution of mononucleosomal DNA fragment lengths from untreated cells of *A. fumigatus* had 2 peaks at 135 nt and 150 nt [16]. The 2 peaks in the distribution of mononucleosomal DNA fragment lengths disappeared after TSA treatment (Fig. 1). In the untreated cells, although the gene bodies of the active and inactive genes and the inactive gene promoters had 2 peaks, the active gene promoters lost the longer peak, suggesting that the nucleosome-wrapped longer DNA in the promoters inhibits high gene expression [16]. However, in the TSA-treated cells, we did not find any difference in the distribution of the mononucleosomal DNA fragment lengths between the active gene bodies and promoters and the inactive gene bodies and promoters (Fig. S5). This finding suggests that the nucleosome-wrapped longer DNA does not inhibit high gene expression in the TSA-treated cells of *A. fumigatus*.

The acetylation level of histones may be much higher in the TSA-treated cells than in the untreated cells. The hyperacetylated histones would be larger than canonical histones in the molecular size. The distributions of the mononucleosomal and dinucleosomal DNA fragment lengths of the TSA-treated cells were significantly different from those of the normal cells (Figs. 1 and 2). Both the mononucleosomal and dinucleosomal DNA fragment lengths of the TSA-treated cells were longer than those of the untreated cells. In addition, the lengths of the mononucleosomal and dinucleosomal DNA fragments that were subjected to a 30-min treatment with MNase were shorter than those subjected to a 15-min treatment with MNase (Figs. 1 and 2). This decrease in fragment lengths indicates digestion of the terminal regions of the nucleosome-bound DNA by MNase during the 15-min treatment.

Between the TSA-treated and untreated cells of *A. fumigatus*, the nucleosome positions were more conserved in the gene promoters than in the gene bodies (Fig. 4). In particular, the nucleosome positions were significantly (*p*-value<0.05) conserved in the gene promoters than in the gene bodies of the down-regulated genes. Generally, the density of nucleosomes in the gene promoters is lower than that in the gene bodies [12], [15], [18]. Thus, the extension of the nucleosomal DNA lengths in the TSA-treated cells influences the nucleosome positions more strongly in the gene bodies than in the gene promoters.

We did not find significant differences between the distributions (Fig. S1) of the squares of Pearson's correlation coefficients for the profiles of the mononucleosome mapping numbers between the TSA-treated and untreated cells at 300 nt downstream and upstream of the 557 TSSs (with >1000 sequence tags). This suggests that some genes have conserved nucleosomes just downstream of the TSS, and some genes have conserved regions upstream of the TSS.

According to the regulated nucleosome mobility model, chromatin is regulated by factors that control the equilibrium between nucleosomes with low and high mobility [27]. In this model, histone acetyltransferase and ATP-dependent nucleosome-remodeling factors (ADNR) induce high mobility of nucleosomes and transcriptional activation. In contrast, histone deacetylase and ADNR induce low mobility of nucleosomes and transcriptional repression. Based on this model, TSA-treated cells have high mobility of nucleosomes and transcriptional activation. However, the number of the up-regulated genes was smaller than that of the down-regulated genes (Table S4). One of the reasons is that although the TSA-treated cells were alive and expressed many genes, the histone-acetylation level of the TSA-treated cells was far different from the normal acetylation level.

Our findings indicate that the histone deacetylase inhibitor TSA influences nucleosome positions by elongation of the nucleosome DNA length. However, most of the nucleosome positions are conserved in the gene promoters between the TSA-treated and untreated cells of the filamentous fungus *Aspergillus fumigatus*, owing to the low density of the nucleosomes of the gene promoters.

## Materials and Methods

### Preparation and sequencing of the paired ends of nucleosomal DNA fragments

In total, 2×10^8^ conidia of *A. fumigatus* Af293 strain were inoculated in 20 ml of 2.4% potato dextrose (PD) medium (Difco Co., Detroit, MI, USA). Cells were grown in the presence of TSA (1 µM) for 24 h at 28°C on a rotary shaker at 160 rpm and then harvested by filtration. MNase digestions were performed as described previously [28]. MNase (TAKARA BIO, Kyoto, Japan) was used at a concentration of 5 U/mg of mycelium for 15 or 30 min at 37°C. We sequenced and mapped the paired ends of the nucleosomal DNA fragments using a massively parallel sequencing platform (Illumina, San Diego, CA, USA). After the chromatin sample had been treated with MNase, the cleavage products were analyzed by agarose gel electrophoresis. We then cut off the mononucleosomal and dinucleosomal DNA fragments separately. Finally, we sequenced both ends (36 bases for the 15-min MNase-treated sample and 76 bases for the 30-min MNase-treated sample) of the DNA fragments using the Illumina Genome Analyzer. Using ELAND (Anthony J. Cox at Illumina), all reads were mapped to the *A. fumigatus* Af293 genome (GenBank accession numbers NC_007194 to NC_007201 correspond to chromosomes 1 to 8), and all uniquely matching read-pairs were retained.

### Identification of transcription start sites of *A. fumigatus*

We inoculated 2×10^8^ conidia of *A. fumigatus* Af293 strain in 20 ml of 2.4% PD medium. Cells were grown for 24 h at 28°C on a rotary shaker at 160 rpm and then harvested by filtration. Total RNAs were isolated from the untreated *A. fumigatus* cells. Then, the 5′-end sequences were determined by using the oligo-capping method [29]. We sequenced 34 bases of the DNA fragments using the Illumina Genome Analyzer. Using ELAND (Anthony J. Cox at Illumina) all reads were mapped to the *A. fumigatus* Af293 genome (GenBank accession numbers NC_007194 to NC_007201 correspond to chromosomes 1 to 8), and all uniquely matching sequences were retained.

### Microarray analysis

The genome sequence and open reading frame predictions for *A. fumigatus* Af293 were obtained from GenBank accession numbers NC_007194 to NC_007201 (chromosomes 1 to 8). A customized high-density oligonucleotide array (Roche NimbleGen, Inc., Madison, WI, USA) was used for the detection of the transcripts in the TSA-treated *A. fumigatus* cells prepared as described above. The cDNA synthesis, hybridization, and scanning were performed by Roche NimbleGen Systems. We compared the results of TSA-treated cells with those of untreated cells (from our previous study) and selected genes that had a more than 2-fold change in expression. We confirmed that all our data is MIAME compliant and that the raw data has been deposited in a MIAME compliant database under accession no. GSE19682.

## Supporting Information

Table S1 Expression levels of the transcriptionally active genes of the TSA-treated cells of *Aspergillus fumigatus*.
(0.13 MB DOC)

Table S2 Expression levels of the transcriptionally inactive genes of the TSA-treated cells of *Aspergillus fumigatus*.
(0.13 MB DOC)

Table S3 Expression levels of the histone-related genes between the TSA-treated and untreated cells.
(0.16 MB DOC)

Table S4 Expression levels of the constant expressed genes between the TSA-treated and untreated cells.
(0.27 MB DOC)

Table S5 Expression levels of the genes with more than 2 fold changes between the TSA-treated and untreated cells.
(0.37 MB DOC)

Figure S1 Boxplots of the squares of Pearson's correlation coefficient between the profiles of the TSA-treated (15-min treatment with MNase) and untreated mononucleosome mapping numbers in the 300 nt downstream and upstream of each of the 557 transcription start sites (>1000 sequence tags).
(0.10 MB PPT)

Figure S2 Mapping numbers of mononucleosomes and transcription start sites of the 50 constant expressed genes between the TSA-treated and untreated cells. Title indicates gene name with the last character “p” or “m”. The “p” indicates that the region between the positions 1 and 1,000 is the promoter and the other region is gene body. The “m” indicates that the region between the position 1,000 downstream from the last position and the last position is the promoter and the other region is gene body. Red and blue indicate the mononucleosome mapping number of the untreated cells and that of the TSA-treated cells respectively. The arrow indicates the region from the translational start to the end. The bars indicate the transcription start sites and the mapping numbers.
(1.76 MB PPT)

Figure S3 Mapping numbers of mononucleosomes and transcription start sites of the 49 down-regulated genes between the TSA-treated and untreated cells. Title indicates gene name with the last character “p” or “m”. The “p” indicates that the region between the positions 1 and 1,000 is the promoter and the other region is gene body. The “m” indicates that the region between the position 1,000 downstream from the last position and the last position is the promoter and the other region is gene body. Red and blue indicate the mononucleosome mapping number of the untreated cells and that of the TSA-treated cells respectively. The arrow indicates the region from the translational start to the end. The bars indicate the transcription start sites and the mapping numbers.
(1.11 MB PPT)

Figure S4 Mapping numbers of mononucleosomes and transcription start sites of the 28 up-regulated genes between the TSA-treated and untreated cells. Title indicates gene name with the last character “p” or “m”. The “p” indicates that the region between the positions 1 and 1,000 is the promoter and the other region is gene body. The “m” indicates that the region between the position 1,000 downstream from the last position and the last position is the promoter and the other region is gene body. Red and blue indicate the mononucleosome mapping number of the untreated cells and that of the TSA-treated cells respectively. The arrow indicates the region from the translational start to the end. The bars indicate the transcription start sites and the mapping numbers.
(0.68 MB PPT)

Figure S5 Histograms of mononucleosomal DNA fragment lengths in the promoters and bodies of transcriptionally active and inactive genes of the TSA-treated cells. We extracted highly expressed (active) genes (not including rRNA genes) and lowly expressed or silent (inactive) genes based on the microarray data of RNAs from the TSA-treated cells of Aspergillus fumigatus.
(0.17 MB PPT)

## References

[1] Igo-Kemenes T, Hörz W, Zachau HG (1982). Chromatin.. Ann Rev Biochem.

[2] Luger K (2006). Dynamic nucleosomes.. Chromosome Res.

[3] Luger K, Mäder AW, Richmond RK, Sargent DF, Richmond TJ (1997). Crystal structure of the nucleosome core particle at 2.8 Å resolution.. Nature.

[4] Millar CB, Grunstein M (2006). Genome-wide patterns of histone modifications in yeast.. Nat Rev Mol Cell Biol.

[5] Lee KK, Workman JL (2007). Histone acetyltransferase complexes: one size doesn't fit all.. Nat Rev Mol Cell Biol.

[6] De Ruijter AJM, Van Gennip AH, Caron HN, Kemp S, Van Kuilenburg ABP (2003). Histone deacetylases (HDACs): characterization of the classical HDAC family.. Biochem J.

[7] Li B, Carey M, Workman JL (2007). The role of chromatin during transcription.. Cell.

[8] Luger K, Richmond TJ (1998). The histone tails of the nucleosome.. Curr Opin Genet Dev.

[9] Sasaki K, Ito T, Nishino N, Khochbin S, Yoshida M (2009). Real-time imaging of histone H4 hyperacetylation in living cells.. Proc Natl Acad Sci USA.

[10] Yoshida M, Horinouchi S, Beppu T (1995). Trichostatin A and trapoxin: novel chemical probes for the role of histone acetylation in chromatin structure and function.. BioEssays.

[11] Dennis JH, Fan H-Y, Reynolds SM, Yuan G, Meldrim JC (2007). Independent and complementary methods for large-scale structural analysis of mammalian chromatin.. Genome Res.

[12] Lee C-K, Shibata Y, Rao B, Strahl BD, Lieb JD (2004). Evidence for nucleosome depletion at active regulatory regions genome-wide.. Nat Genet.

[13] Lee W, Tillo D, Bray N, Morse RH, Davis RW (2007). A high-resolution atlas of nucleosome occupancy in yeast.. Nat Genet.

[14] Mavrich TN, Jiang C, Ioshikhes IP, Li X, Venters BJ (2008). Nucleosome organization in the *Drosophila* genome.. Nature.

[15] Nishida H, Suzuki T, Kondo S, Miura H, Fujimura Y (2006). Histone H3 acetylated at lysine 9 in promoter is associated with low nucleosome density in the vicinity of transcription start site in human cell.. Chromosome Res.

[16] Nishida H, Motoyama T, Yamamoto S, Aburatani H, Osada H (2009). Genome-wide maps of mono- and di-nucleosomes of *Aspergillus fumigatus*.. Bioinformatics.

[17] Schones DE, Cui K, Cuddapah S, Roh T-Y, Barski A (2008). Dynamic regulation of nucleosome positioning in the human genome.. Cell.

[18] Segal E, Fondufe-Mittendorf Y, Chen L, Thåström AC, Field Y (2006). A genomic code for nucleosome positioning.. Nature.

[19] Shivaswamy S, Bhinge A, Zhao Y, Jones S, Hirst M (2008). Dynamic remodeling of individual nucleosomes across a eukaryotic genome in response to transcriptional perturbation.. PLoS Biol.

[20] Song JS, Liu X, Liu XS, He X (2008). A high-resolution map of nucleosome positioning on a fission yeast centromere.. Genome Res.

[21] Valouev A, Ichikawa J, Tonthat T, Stuart J, Ranade S (2008). A high-resolution, nucleosome position map of *C. elegans* reveals a lack of universal sequence-dictated positioning.. Genome Res.

[22] Yuan G-C, Liu Y-J, Dion MF, Slack MD, Wu LF (2005). Genome-scale identification of nucleosome positions in *S. cerevisiae*.. Science.

[23] Nishida H (2009). Calculation of the ratio of the mononucleosome mapping number to the dinucleosome mapping number for each nucleotide position in the *Aspergillus fumigatus* genome.. Open Access Bioinformatics.

[24] Graessle S, Dangl M, Haas H, Mair K, Trojer P (2000). Characterization of two putative histone deacetylase genes from *Aspergillus nidulans*.. Biochim Biophys Acta.

[25] Nishida H (2009). Evolutionary conservation levels of subunits of histone-modifying protein complexes in fungi.. Comp Funct Genomics.

[26] Tribus M, Bauer I, Galehr J, Rieser G, Trojer P (2010). A novel motif fungal class 1 histone deacetylases is essential for growth and development of *Aspergillus*.. Mol Biol Cell.

[27] Cosgrove MS, Boeke JD, Wolberger C (2004). Regulated nucleosome mobility and the histone code.. Nat Struct Mol Biol.

[28] Gonzalez R, Scazzocchio C (1997). A rapid method for chromatin structure analysis in the filamentous fungus *Aspergillus nidulans*.. Nucleic Acids Res.

[29] Suzuki Y, Sugano S (2003). Construction of a full-length enriched and a 5′-end enriched cDNA library using the oligo-capping method.. Methods Mol Biol.

