# Supplementary material for: Genome-Wide Maps of Mononucleosomes and Dinucleosomes Containing Hyperacetylated Histones of Aspergillus fumigatus
Source: PLoS One. 2010 Mar 26;5(3):e9916. doi: 10.1371/journal.pone.0009916 (PMC2845647; doi:10.1371/journal.pone.0009916)
Supplement: Table S1 — Expression levels of the transcriptionally active genes of the TSA-treated cells of Aspergillus fumigatus. (0.13 MB DOC) [file pone.0009916.s001.doc]

| Supplementary Table S1. Expression levels of the transcriptionally active genes of the TSA-treated cells of *Aspergillus fumigatus*. | | | | | | | | | |
| --- | --- | --- | --- | --- | --- | --- | --- | --- | --- |
| Gene | Intensity (1) | Intensity (2) | Total intensities | Length (nt) | Chromosome | Gene body | | Strand | Annotation |
| Afu1g02550 | 36171.5349 | 38232.478 | 74404.0129 | 1753 | 1 | 744583 | 746335 | - | tubulin alpha-1 subunit |
| Afu1g03630 | 36660.9908 | 44532.0653 | 81193.0561 | 753 | 1 | 1056979 | 1057731 | + | GPI anchored protein, putative |
| Afu1g04320 | 44135.2249 | 50819.8271 | 94955.052 | 655 | 1 | 1226288 | 1226942 | + | ribosomal protein S8.e |
| Afu1g05080 | 46443.3227 | 49074.2671 | 95517.5898 | 1075 | 1 | 1454060 | 1455134 | + | ribosomal protein P0 |
| Afu1g05390 | 54933.8927 | 53111.665 | 108045.558 | 1186 | 1 | 1548016 | 1549201 | - | mitochondrial ADP,ATP carrier protein (Ant),putative |
| Afu1g05790 | 37594.2831 | 36961.7831 | 74556.0662 | 634 | 1 | 1670236 | 1670869 | - | GPI anchored protein, putative |
| Afu1g06390 | 55706.5001 | 60800.5408 | 116507.041 | 1562 | 1 | 1831900 | 1833461 | + | translation elongation factor EF-1 alpha subunit, putative |
| Afu1g12890 | 41501.2168 | 40713.5331 | 82214.7499 | 1141 | 1 | 3406534 | 3407674 | + | 60s ribosomal protein l1 |
| Afu1g14200 | 36378.6114 | 38261.834 | 74640.4454 | 1688 | 1 | 3794154 | 3795841 | - | mitochondrial processing peptidase beta subunit,putative |
| Afu2g04620 | 36523.6393 | 38002.2993 | 74525.9386 | 2197 | 2 | 1258486 | 1260682 | - | ER Hsp70 chaperone BiP, putative |
| Afu2g09960 | 45330.8824 | 44133.7754 | 89464.6578 | 2224 | 2 | 2551872 | 2554095 | - | mitochondrial Hsp70 chaperone (Ssc70), putative |
| Afu2g11520 | 54116.1606 | 52933.773 | 107049.934 | 1795 | 2 | 2968967 | 2970761 | - | MFS monosaccharide transporter, putative |
| Afu2g11850 | 43412.9432 | 46449.3227 | 89862.2659 | 1540 | 2 | 3060821 | 3062360 | + | large subunit ribosomal protein L3 |
| Afu2g13530 | 43888.2153 | 44419.2831 | 88307.4984 | 2919 | 2 | 3518248 | 3521166 | + | translation elongation factor EF-2 subunit,putative |
| Afu2g17110 | 40060.4462 | 39171.7831 | 79232.2293 | 2763 | 2 | 4568158 | 4570920 | + | cell division control protein Cdc48 |
| Afu2g17840 | 40497.6858 | 37643.7994 | 78141.4852 | 1628 | 2 | 4743947 | 4745574 | + | MFS transporter, putative |
| Afu3g00270 | 50780.433 | 52075.7014 | 102856.134 | 1407 | 3 | 56302 | 57708 | - | cell wall glucanase, putative |
| Afu3g07640 | 51507.2389 | 52481.3076 | 103988.547 | 3109 | 3 | 1930419 | 1933527 | + | plasma membrane H+-ATPase |
| Afu3g08110 | 56358.3448 | 56060.5802 | 112418.925 | 1848 | 3 | 2077251 | 2079098 | + | cell wall protein, putative |
| Afu3g08160 | 46133.5581 | 40908.4315 | 87041.9896 | 1537 | 3 | 2094223 | 2095759 | - | eukaryotic translation initiation factor eIF4A,putative |
| Afu3g11740 | 38789.0771 | 35281.1253 | 74070.2024 | 2810 | 3 | 3081634 | 3084443 | + | conserved hypothetical protein |
| Afu4g06670 | 40162.9157 | 44745.7833 | 84908.699 | 813 | 4 | 1724024 | 1724836 | - | allergen Asp F7 |
| Afu4g06820 | 37186.7118 | 41965.5891 | 79152.3009 | 1324 | 4 | 1758702 | 1760025 | + | related to sporulation-specific gene SPS2,putative |
| Afu4g07360 | 43031.2236 | 47951.962 | 90983.1856 | 2520 | 4 | 1908017 | 1910536 | - | 5-methyltetrahydropteroyltriglutamate--homocysteine  S-methyltransferase |
| Afu4g09350 | 37411.2558 | 36558.8892 | 73970.145 | 756 | 4 | 2446436 | 2447191 | + | hypothetical protein |
| Afu4g09360 | 56526.7041 | 55827.2328 | 112353.937 | 474 | 4 | 2446685 | 2447158 | - | ATP synthase proteolipid P2, putative |
| Afu4g11340 | 43825.5473 | 41465.0608 | 85290.6081 | 1772 | 4 | 2973300 | 2975071 | - | saccharopine dehydrogenase |
| Afu4g11550 | 39494.6887 | 40263.8798 | 79758.5685 | 1150 | 4 | 3041618 | 3042767 | - | hypothetical protein |
| Afu4g13120 | 36191.8041 | 37308.9646 | 73500.7687 | 1749 | 4 | 3431115 | 3432863 | - | glutamine synthetase |
| Afu5g01650 | 38388.1143 | 43726.2557 | 82114.37 | 795 | 5 | 420067 | 420861 | + | bZIP transcription factor (JlbA), putative |
| Afu5g03020 | 50360.918 | 56445.1704 | 106806.088 | 1390 | 5 | 806662 | 808051 | + | 60s ribosomal protein l2 |
| Afu5g03760 | 55941.9776 | 58573.8746 | 114515.852 | 2808 | 5 | 1009794 | 1012601 | - | class III chitinase ChiA1 |
| Afu5g04230 | 42017.128 | 39414.1032 | 81431.2312 | 1895 | 5 | 1127276 | 1129170 | - | citrate synthase, eukaryotic |
| Afu5g08830 | 49552.0222 | 48173.4593 | 97725.4815 | 2389 | 5 | 2265787 | 2268175 | + | HEX1 |
| Afu5g10010 | 54793.2657 | 53956.7192 | 108749.985 | 801 | 5 | 2583303 | 2584103 | + | conserved hypothetical protein |
| Afu5g10550 | 46397.9057 | 44465.7831 | 90863.6888 | 2080 | 5 | 2700129 | 2702208 | - | ATP synthase F1, beta subunit, putative |
| Afu5g10560 | 37857.2831 | 38248.0331 | 76105.3162 | 756 | 5 | 2703448 | 2704203 | - | cytochrome c oxidase subunit V |
| Afu6g04570 | 39821.0695 | 42181.1412 | 82002.2107 | 1864 | 6 | 1060589 | 1062452 | + | elongation factor 1-gamma 2 |
| Afu6g04740 | 43622.3644 | 48194.4435 | 91816.8079 | 1335 | 6 | 1113100 | 1114434 | - | actin, putative |
| Afu6g10660 | 39166.6495 | 44137.2774 | 83303.9269 | 1523 | 6 | 2635628 | 2637150 | + | ATP-citrat-lyase |
| Afu6g12930 | 33248.9517 | 40749.1683 | 73998.12 | 2867 | 6 | 3262693 | 3265559 | + | aconitate hydratase, mitochondrial |
| Afu6g14090 | 39074.9753 | 37065.2782 | 76140.2535 | 1045 | 6 | 3593051 | 3594095 | + | CFEM domain protein, putative |
| Afu7g00170 | 38799.5535 | 38056.1018 | 76855.6553 | 1362 | 7 | 42071 | 43432 | + | dimethylallyl tryptophan synthase-related |
| Afu7g01490 | 44955.5331 | 48364.8331 | 93320.3662 | 1876 | 7 | 388607 | 390482 | + | MFS peptide transporter, putative |
| Afu7g02140 | 45653.428 | 48459.0146 | 94112.4426 | 411 | 7 | 573928 | 574338 | + | RPS24 |
| Afu7g05660 | 40312.2636 | 38731.5331 | 79043.7967 | 3311 | 7 | 1376127 | 1379437 | + | elongation factor EF-3, putative |
| Afu8g01690 | 41076.033 | 38582.9479 | 79658.9809 | 478 | 8 | 444779 | 445256 | + | hypothetical protein |
| Afu8g03930 | 46634.7837 | 43563.5331 | 90198.3168 | 2292 | 8 | 839316 | 841607 | + | Hsp70 chaperone (HscA), putative |
| Afu8g05320 | 40659.8136 | 44514.6927 | 85174.5063 | 2097 | 8 | 1239645 | 1241741 | + | mitochondrial F1 ATPase subunit alpha, putative |
| Afu8g05610 | 43189.82 | 43983.8346 | 87173.6546 | 2084 | 8 | 1318148 | 1320231 | + | cell wall glucanase (Scw11), putative |
