# Supplementary material for: Genome-Wide Maps of Mononucleosomes and Dinucleosomes Containing Hyperacetylated Histones of Aspergillus fumigatus
Source: PLoS One. 2010 Mar 26;5(3):e9916. doi: 10.1371/journal.pone.0009916 (PMC2845647; doi:10.1371/journal.pone.0009916)
Supplement: Table S2 — Expression levels of the transcriptionally inactive genes of the TSA-treated cells of Aspergillus fumigatus. (0.13 MB DOC) [file pone.0009916.s002.doc]

| Supplementary Table S2. Expression levels of the transcriptionally inactive genes of the TSA-treated cells of *Aspergillus fumigatus*. | | | | | | | | | |
| --- | --- | --- | --- | --- | --- | --- | --- | --- | --- |
| Gene | Intensity (1) | Intensity (2) | Total intensities | Length (nt) | Chromosome | Gene body | | Strand | Annotation |
| Afu1g00140 | 1073.5926 | 1064.5711 | 2138.1637 | 2288 | 1 | 13021 | 15308 | + | transposase |
| Afu1g03240 | 1451.9155 | 1315.2197 | 2767.1352 | 237 | 1 | 944467 | 944703 | + | hypothetical protein |
| Afu1g04730 | 1556.4671 | 1540.4828 | 3096.9499 | 767 | 1 | 1342452 | 1343218 | + | endoglucanase, putative |
| Afu1g09340 | 1015.8351 | 911.0996 | 1926.9347 | 471 | 1 | 2424179 | 2424649 | + | hypothetical protein |
| Afu1g10120 | 1206.3953 | 1238.5498 | 2444.9451 | 1296 | 1 | 2608548 | 2609843 | + | ABC transporter permease protein |
| Afu1g11270 | 1117.1335 | 1210.756 | 2327.8895 | 1608 | 1 | 2973374 | 2974981 | - | conserved hypothetical protein |
| Afu1g13810 | 1302.95 | 1527.6995 | 2830.6495 | 289 | 1 | 3687664 | 3687952 | + | hypothetical protein |
| Afu1g17300 | 1219.1286 | 1185.347 | 2404.4756 | 1613 | 1 | 4726822 | 4728434 | + | hypothetical protein |
| Afu2g00600 | 1466.6867 | 1466.8848 | 2933.5715 | 597 | 2 | 127896 | 128492 | + | conserved hypothetical protein |
| Afu2g04180 | 1147.7297 | 1023.8516 | 2171.5813 | 150 | 2 | 1160599 | 1160748 | - | hypothetical protein |
| Afu2g04420 | 890.254 | 1027.2782 | 1917.5322 | 2265 | 2 | 1213702 | 1215966 | - | hypothetical protein |
| Afu2g05160 | 1665.9141 | 1452.4371 | 3118.3512 | 159 | 2 | 1427934 | 1428092 | - | hypothetical protein |
| Afu2g09440 | 1536.7831 | 1461.6169 | 2998.4 | 353 | 2 | 2411439 | 2411791 | + | acetyl xylan esterase |
| Afu2g09880 | 1548.765 | 1332.0837 | 2880.8487 | 490 | 2 | 2524927 | 2525416 | + | hypothetical protein |
| Afu2g09970 | 1395.3323 | 1293.4737 | 2688.806 | 393 | 2 | 2554705 | 2555097 | - | hypothetical protein |
| Afu2g13025 | 682.2117 | 664.7831 | 1346.9948 | 849 | 2 | 3343334 | 3344182 | + | conserved hypothetical protein |
| Afu2g13300 | 854.7831 | 980.7831 | 1835.5662 | 1736 | 2 | 3435489 | 3437224 | - | conserved hypothetical protein |
| Afu2g13900 | 1601.0213 | 1483.9879 | 3085.0092 | 478 | 2 | 3628990 | 3629467 | + | multidrug resistance protein MDR |
| Afu3g01470 | 1533.0331 | 1626.3132 | 3159.3463 | 1013 | 3 | 372686 | 373698 | + | hypothetical protein |
| Afu3g03270 | 1173.2759 | 1378.3296 | 2551.6055 | 862 | 3 | 874449 | 875310 | + | isochorismatase family hydrolase, putative |
| Afu3g03540 | 1435.3347 | 1592.6838 | 3028.0185 | 2664 | 3 | 947249 | 949912 | + | polyketide synthase, putative |
| Afu3g09510 | 1549.047 | 1402.7221 | 2951.7691 | 1578 | 3 | 2418354 | 2419931 | + | 3-oxoacyl-(acyl-carrier-protein) reductase |
| Afu3g13130 | 1487.002 | 1476.5366 | 2963.5386 | 739 | 3 | 3486744 | 3487482 | - | HHE domain protein |
| Afu3g14580 | 1083.0209 | 1198.4432 | 2281.4641 | 531 | 3 | 3863777 | 3864307 | - | deoxycytidine triphosphate deaminase, putative |
| Afu4g01430 | 1528.8901 | 1395.9364 | 2924.8265 | 347 | 4 | 374980 | 375326 | + | hypothetical protein |
| Afu4g01630 | 1003.1359 | 1089.5029 | 2092.6388 | 3990 | 4 | 433689 | 437678 | - | reverse transcriptase, putative |
| Afu4g02740 | 1606.2036 | 1492.6341 | 3098.8377 | 484 | 4 | 762040 | 762523 | + | hypothetical protein |
| Afu4g06650 | 1270.3499 | 995.5225 | 2265.8724 | 1619 | 4 | 1719359 | 1720977 | - | hypothetical protein |
| Afu4g06660 | 1592.7823 | 1515.2831 | 3108.0654 | 538 | 4 | 1721926 | 1722463 | - | hypothetical protein |
| Afu4g14530 | 901.5332 | 1008.6299 | 1910.1631 | 678 | 4 | 3831931 | 3832608 | + | theta class glutathione S-transferase |
| Afu4g14690 | 1284.6931 | 1413.9626 | 2698.6557 | 945 | 4 | 3869297 | 3870241 | - | hypothetical protein |
| Afu5g02960 | 1159.3981 | 1240.8461 | 2400.2442 | 446 | 5 | 790739 | 791184 | - | hypothetical protein |
| Afu5g06850 | 1208.3722 | 1394.0331 | 2602.4053 | 1881 | 5 | 1685289 | 1687169 | + | hypothetical protein |
| Afu5g10270 | 1449.0331 | 1336.9848 | 2786.0179 | 606 | 5 | 2631019 | 2631624 | + | heat shock protein, HSP20 family |
| Afu5g11060 | 1508.8422 | 1478.2063 | 2987.0485 | 1426 | 5 | 2832669 | 2834094 | - | WD repeat protein |
| Afu5g11950 | 1334.0155 | 1600.0554 | 2934.0709 | 738 | 5 | 3090620 | 3091357 | - | Ubiquitin carboxyl-terminal hydrolase, family 1superfamily |
| Afu5g13960 | 1601.1645 | 1474.4847 | 3075.6492 | 448 | 5 | 3652550 | 3652997 | - | hypothetical protein |
| Afu5g14840 | 1512.3835 | 1363.13 | 2875.5135 | 3406 | 5 | 3834455 | 3837860 | - | hypothetical protein |
| Afu5g14990 | 1341.6784 | 1163.3638 | 2505.0422 | 783 | 5 | 3878797 | 3879579 | - | hypothetical protein |
| Afu6g11750 | 1175.2801 | 1200.9464 | 2376.2265 | 1722 | 6 | 2923673 | 2925394 | + | C6 transcription factor, putative |
| Afu6g11760 | 1447.159 | 1535.147 | 2982.306 | 333 | 6 | 2925442 | 2925774 | + | hypothetical protein |
| Afu6g11770 | 1614.6056 | 1459.9324 | 3074.538 | 1070 | 6 | 2926410 | 2927479 | + | hypothetical protein |
| Afu6g14690 | 1290.1893 | 1341.8982 | 2632.0875 | 1332 | 6 | 3771090 | 3772421 | + | putative transposase |
| Afu7g06140 | 1259.5626 | 1452.585 | 2712.1476 | 2366 | 7 | 1507248 | 1509613 | - | beta-D-glucoside glucohydrolase |
| Afu8g02230 | 1492.3334 | 1319.8896 | 2812.223 | 764 | 8 | 579643 | 580406 | + | deliriumA |
| Afu8g02270 | 1375.3979 | 1442.7734 | 2818.1713 | 891 | 8 | 608288 | 609178 | + | dihydrodipicolinate synthetase family protein |
| Afu8g05040 | 1266.8001 | 1574.192 | 2840.9921 | 951 | 8 | 1174836 | 1175786 | - | dihydrodipicolinate synthetase family protein |
| Afu8g05080 | 1597.7424 | 1404.0384 | 3001.7808 | 1488 | 8 | 1181984 | 1183471 | + | hypothetical protein |
| Afu8g06200 | 1242.2264 | 1085.4928 | 2327.7192 | 504 | 8 | 1479155 | 1479658 | + | hypothetical protein |
| Afu8g06860 | 1356.2433 | 1684.8436 | 3041.0869 | 855 | 8 | 1679129 | 1679983 | - | conserved hypothetical protein |
