# Supplementary material for: Genome-Wide Maps of Mononucleosomes and Dinucleosomes Containing Hyperacetylated Histones of Aspergillus fumigatus
Source: PLoS One. 2010 Mar 26;5(3):e9916. doi: 10.1371/journal.pone.0009916 (PMC2845647; doi:10.1371/journal.pone.0009916)
Supplement: Table S3 — Expression levels of the histone-related genes between the TSA-treated and untreated cells. (0.16 MB DOC) [file pone.0009916.s003.doc]

| Supplementary Table S3. Expression levels of the histone-related genes between the TSA-treated and untreated cells. | | | | | | | | | |  |
| --- | --- | --- | --- | --- | --- | --- | --- | --- | --- | --- |
| Gene | Intensity (w/o TSA) | Intensity (with TSA) | Fold changes | Down/Up | Length | Chromosome | Gene body | | Strand | Annotation |
| Afu1g05490 | 20556.8328 | 19419.0507 | 1.05859103 | DOWN | 3843 | 1 | 1568506 | 1572348 | + | histone deacetylase complex  subunit (Hos4),putative |
|  | 18438.8572 | 18378.706 | 1.00327287 | DOWN |  |  |  |  |  |
| Afu1g06720 | 6024.8988 | 6535.0801 | 1.08467882 | UP | 1722 | 1 | 1923127 | 1924848 | - | histone acetyltransferase  Spt10, putative |
|  | 5972.6931 | 5709.1839 | 1.04615532 | DOWN |  |  |  |  |  |
| Afu1g10540 | 6180.7446 | 7361.2946 | 1.19100449 | UP | 1918 | 1 | 2742783 | 2744700 | + | SIR2 family histone  deacetylase (Hst4),putative |
|  | 6913.9901 | 6583.8137 | 1.05014972 | DOWN |  |  |  |  |  |
| Afu1g13780 | 2736.995 | 2624.712 | 1.04277917 | DOWN | 438 | 1 | 3680611 | 3681048 | - | histone H4 |
|  | 2521.5742 | 2606.1675 | 1.03354781 | UP |  |  |  |  |  |
| Afu1g13790 | 11669.2916 | 13006.9305 | 1.11462897 | UP | 593 | 1 | 3681876 | 3682468 | + | histone H3 |
|  | 12726.839 | 13394.2475 | 1.05244103 | UP |  |  |  |  |  |
| Afu2g03390 | 10873.0481 | 16679.1856 | 1.53399355 | UP | 2248 | 2 | 893605 | 895852 | - | histone deacetylase RpdA |
|  | 9092.5397 | 17127.6633 | 1.88370509 | UP |  |  |  |  |  |
| Afu2g03810 | 9578.8392 | 10918.8531 | 1.13989314 | UP | 1464 | 2 | 1011228 | 1012691 | - | histone deacetylase HosA |
|  | 9414.2951 | 10879.1882 | 1.15560306 | UP |  |  |  |  |  |
| Afu2g05530 | 6130.2692 | 5873.3375 | 1.04374543 | DOWN | 1636 | 2 | 1544205 | 1545840 | - | histone acetyltransferase  (Esa1), putative |
|  | 6119.9267 | 6115.7444 | 1.00068386 | DOWN |  |  |  |  |  |
| Afu2g05900 | 6053.2628 | 5217.0721 | 1.16027969 | DOWN | 1332 | 2 | 1660650 | 1661981 | + | SIR2 family histone  deacetylase, putative |
|  | 5529.1652 | 4796.153 | 1.15283336 | DOWN |  |  |  |  |  |
| Afu2g06110 | 12313.9123 | 12186.116 | 1.01048704 | DOWN | 1465 | 2 | 1733986 | 1735450 | + | chromatin remodeling  and histoneacetyltransferase  complexes subunit (Arp4) putative |
|  | 12529.1879 | 11415.4637 | 1.09756276 | DOWN |  |  |  |  |  |
| Afu2g12030 | 8333.3996 | 10771.3206 | 1.29254819 | UP | 1873 | 2 | 3094723 | 3096595 | - | histone acetyltransferase  type b catalyticsubunit, putative |
|  | 8912.3754 | 9673.2158 | 1.08536898 | UP |  |  |  |  |  |
| Afu2g13860 | 2442.5806 | 2350.0072 | 1.03939282 | DOWN | 557 | 2 | 3619593 | 3620149 | + | histone H4 |
|  | 2323.3624 | 2638.2004 | 1.13550964 | UP |  |  |  |  |  |
| Afu3g00520 | 8338.9885 | 8516.2547 | 1.02125752 | UP | 1257 | 3 | 113361 | 114617 | + | SIR2 family histone  deacetylase, putative |
|  | 9149.2847 | 8651.8174 | 1.05749859 | DOWN |  |  |  |  |  |
| Afu3g05350 | 19456.3284 | 20090.2831 | 1.03258347 | UP | 576 | 3 | 1290284 | 1290859 | - | histone H2B |
|  | 17743.0331 | 22489.6768 | 1.26752155 | UP |  |  |  |  |  |
| Afu3g05360 | 19786.3213 | 18994.6047 | 1.04168113 | DOWN | 924 | 3 | 1291520 | 1292443 | + | histone H2A |
|  | 18248.281 | 20324.3499 | 1.11376792 | UP |  |  |  |  |  |
| Afu3g06070 | 4963.9328 | 4117.4221 | 1.2055924 | DOWN | 1193 | 3 | 1494205 | 1495397 | + | histone H1 |
|  | 4693.4566 | 4470.91 | 1.04977658 | DOWN |  |  |  |  |  |
| Afu3g09610 | 4824.0098 | 4493.3847 | 1.07358041 | DOWN | 1068 | 3 | 2442714 | 2443781 | - | histone acetyltransferase  (MysT1), putative |
|  | 5175.9835 | 4744.8734 | 1.09085808 | DOWN |  |  |  |  |  |
| Afu4g04290 | 9036.458 | 8284.1595 | 1.09081169 | DOWN | 3576 | 4 | 1201785 | 1205360 | - | histone deacetylase  family protein |
|  | 8794.3949 | 7032.6877 | 1.25050269 | DOWN |  |  |  |  |  |
| Afu4g08295 | 4802.8494 | 5240.7369 | 1.09117244 | UP | 786 | 4 | 2144623 | 2145408 | + | histone h1.3., putative |
|  | 4865.5071 | 4599.3334 | 1.05787223 | DOWN |  |  |  |  |  |
| Afu4g09820 | 10099.8824 | 10757.6284 | 1.06512412 | UP | 878 | 4 | 2549834 | 2550711 | + | histone acetyltransferase  subunit (Yaf9),putative |
|  | 11148.3584 | 10393.7621 | 1.07260088 | DOWN |  |  |  |  |  |
| Afu4g10660 | 2737.4722 | 2465.5331 | 1.11029627 | DOWN | 1374 | 4 | 2778843 | 2780216 | + | histone acetylase complex  subunit MRG15-2 |
|  | 2468.6288 | 2697.0331 | 1.09252274 | UP |  |  |  |  |  |
| Afu4g10910 | 14190.0331 | 13560.0331 | 1.04646006 | DOWN | 3336 | 4 | 2846152 | 2849487 | - | histone acetyltransferase,  putative |
|  | 14068.6523 | 11743.0933 | 1.19803632 | DOWN |  |  |  |  |  |
| Afu4g12120 | 6709.2208 | 6797.5073 | 1.01315898 | UP | 1737 | 4 | 3181274 | 3183010 | + | SIR2 family histone  deacetylase, putative |
|  | 6434.4127 | 7075.6933 | 1.0996642 | UP |  |  |  |  |  |
| Afu4g12650 | 5871.12 | 6600.8756 | 1.12429581 | UP | 1405 | 4 | 3316322 | 3317726 | - | histone acetyltransferase  (Gcn5), putative |
|  | 5980.0331 | 6007.2831 | 1.00455683 | UP |  |  |  |  |  |
| Afu5g01950 | 14533.9897 | 15908.8662 | 1.09459732 | UP | 820 | 5 | 499510 | 500329 | - | histone H2A |
|  | 13999.3419 | 15821.9628 | 1.13019333 | UP |  |  |  |  |  |
| Afu5g01980 | 5158.9064 | 5996.2814 | 1.16231638 | UP | 2844 | 5 | 506275 | 509118 | + | histone deacetylase  hda1 |
|  | 5472.0664 | 5796.3495 | 1.05926154 | UP |  |  |  |  |  |
| Afu5g02570 | 13767.0767 | 16399.1021 | 1.19118259 | UP | 11895 | 5 | 665470 | 677364 | + | histone acetylase complex  subunit Paf400,putative |
|  | 15382.2819 | 14678.3656 | 1.04795604 | DOWN |  |  |  |  |  |
| Afu5g04120 | 4683.2831 | 4367.1794 | 1.07238166 | DOWN | 1146 | 5 | 1092632 | 1093777 | + | SIR2 family histone  deacetylase, putative |
|  | 5367.7831 | 4254.4694 | 1.26168097 | DOWN |  |  |  |  |  |
| Afu5g06140 | 7790.6043 | 10153.5558 | 1.30330786 | UP | 1725 | 5 | 1466444 | 1468168 | + | histone acetyltransferase,  putative |
|  | 8491.7645 | 9704.0992 | 1.14276594 | UP |  |  |  |  |  |
| Afu6g04660 | 4811.888 | 4406.0356 | 1.09211283 | DOWN | 617 | 6 | 1086108 | 1086724 | - | histone H3 variant,  putative |
|  | 4399.8264 | 4376.6678 | 1.00529138 | DOWN |  |  |  |  |  |
| Afu6g09210 | 5085.8537 | 4448.6004 | 1.14324804 | DOWN | 1033 | 6 | 2185515 | 2186547 | + | SIR2 family histone  deacetylase, putative |
|  | 5655.111 | 4254.326 | 1.32926132 | DOWN |  |  |  |  |  |
