# Supplementary material for: Genome-Wide Maps of Mononucleosomes and Dinucleosomes Containing Hyperacetylated Histones of Aspergillus fumigatus
Source: PLoS One. 2010 Mar 26;5(3):e9916. doi: 10.1371/journal.pone.0009916 (PMC2845647; doi:10.1371/journal.pone.0009916)
Supplement: Table S4 — Expression levels of the constant expressed genes between the TSA-treated and untreated cells. (0.27 MB DOC) [file pone.0009916.s004.doc]

| Supplementary Table S4. Expression levels of the constant expressed genes between the TSA-treated and untreated cells. | | | | | | | | | |  |  |
| --- | --- | --- | --- | --- | --- | --- | --- | --- | --- | --- | --- |
| Gene | Intensity (w/o TSA) | Intensity (with TSA) | Hold changes | Down/up | Mean of changes | Length (nt) | Chromosome | Gene body | | Strand | Annotation |
| Afu1g02100 | 3707.2015 | 3707.9981 | 1.00021488 | UP | 1.00770453 | 4366 | 1 | 616953 | 621318 | + | hypothetical protein |
|  | 3661.9222 | 3717.5621 | 1.01519418 | UP |  |  |  |  |  |  |
| Afu1g07670 | 15606.6403 | 15603.8815 | 1.0001768 | DOWN | 1.00550835 | 2145 | 1 | 2163931 | 2166075 | - | conserved hypothetical  protein |
|  | 14749.5302 | 14909.4136 | 1.0108399 | UP |  |  |  |  |  |  |
| Afu1g16020 | 3964.5831 | 3941.4246 | 1.00587567 | DOWN | 1.00668273 | 844 | 1 | 4356086 | 4356929 | + | hypothetical protein |
|  | 3717.3617 | 3745.204 | 1.0074898 | UP |  |  |  |  |  |  |
| Afu1g16080 | 3417.2481 | 3402.6151 | 1.00430052 | DOWN | 1.00476624 | 720 | 1 | 4374579 | 4375298 | - | hypothetical protein |
|  | 3445.9089 | 3463.9378 | 1.00523197 | UP |  |  |  |  |  |  |
| Afu1g16960 | 4500.1005 | 4503.997 | 1.00086587 | UP | 1.00811211 | 669 | 1 | 4633231 | 4633899 | - | hypothetical protein |
|  | 4428.9767 | 4496.9985 | 1.01535836 | UP |  |  |  |  |  |  |
| Afu2g01500 | 3987.6574 | 3985.8852 | 1.00044462 | DOWN | 1.00280603 | 4581 | 2 | 354415 | 358995 | - | ABC bile acid transporter,  putative |
|  | 4112.2831 | 4133.5331 | 1.00516745 | UP |  |  |  |  |  |  |
| Afu2g03480 | 7310.9253 | 7352.7831 | 1.00572538 | UP | 1.00552665 | 2613 | 2 | 916189 | 918801 | - | conserved hypothetical  protein |
|  | 7315.059 | 7354.0331 | 1.00532793 | UP |  |  |  |  |  |  |
| Afu2g06180 | 9273.1182 | 9209.2405 | 1.00693626 | DOWN | 1.00808204 | 3337 | 2 | 1756129 | 1759465 | + | hypothetical protein |
|  | 9211.2831 | 9296.2831 | 1.00922781 | UP |  |  |  |  |  |  |
| Afu2g06200 | 9749.2992 | 9615.7582 | 1.01388772 | DOWN | 1.00769867 | 1438 | 2 | 1762681 | 1764118 | + | conserved hypothetical  protein |
|  | 10144.8484 | 10129.5566 | 1.00150962 | DOWN |  |  |  |  |  |  |
| Afu2g06330 | 9474.1368 | 9398.0314 | 1.00809802 | DOWN | 1.00768828 | 2355 | 2 | 1817718 | 1820072 | + | ubiquitin C-terminal hydrolase,  putative |
|  | 10223.1865 | 10149.3142 | 1.00727855 | DOWN |  |  |  |  |  |  |
| Afu2g10650 | 3763.9827 | 3765.7931 | 1.00048098 | UP | 1.00383077 | 910 | 2 | 2725087 | 2725996 | + | enoyl-CoA hydratase |
|  | 3928.2714 | 3900.2653 | 1.00718056 | DOWN |  |  |  |  |  |  |
| Afu2g11110 | 9643.956 | 9750.9274 | 1.01109207 | UP | 1.00570443 | 3877 | 2 | 2845207 | 2849083 | - | nuclear condensin complex  subunit Smc2,putative |
|  | 9090.9623 | 9093.8423 | 1.0003168 | UP |  |  |  |  |  |  |
| Afu2g14905 | 16481.1704 | 16564.5158 | 1.00505701 | UP | 1.00466088 | 404 | 2 | 3926842 | 3927245 | - | NADH-ubiquinone oxidoreductase  12 kda subunit,putative |
|  | 16744.514 | 16673.4059 | 1.00426476 | DOWN |  |  |  |  |  |  |
| Afu3g01150 | 2751.412 | 2766.7831 | 1.00558662 | UP | 1.00839457 | 648 | 3 | 268275 | 268922 | + | GPI anchored cell wall protein,  putative |
|  | 2670.8721 | 2641.2831 | 1.01120251 | DOWN |  |  |  |  |  |  |
| Afu3g02800 | 3683.0331 | 3709.7831 | 1.00726304 | UP | 1.00663085 | 684 | 3 | 742040 | 742723 | - | arylesterase/monoxygenase,  putative |
|  | 3521.7831 | 3500.7831 | 1.00599866 | DOWN |  |  |  |  |  |  |
| Afu3g03080 | 2931.0331 | 2914.6812 | 1.00561018 | DOWN | 1.00541501 | 947 | 3 | 824479 | 825425 | + | endo-1,3(4)-beta-glucanase,  putative |
|  | 3038.0589 | 3022.2831 | 1.00521983 | DOWN |  |  |  |  |  |  |
| Afu3g03800 | 7017.6584 | 7038.4132 | 1.00295751 | UP | 1.00225831 | 553 | 3 | 1096036 | 1096588 | - | hypothetical protein |
|  | 6628.6596 | 6638.9944 | 1.00155911 | UP |  |  |  |  |  |  |
| Afu3g03850 | 3088.9767 | 3080.5952 | 1.00272074 | DOWN | 1.00294308 | 938 | 3 | 1104755 | 1105692 | - | hypothetical protein |
|  | 3171.2226 | 3161.216 | 1.00316543 | DOWN |  |  |  |  |  |  |
| Afu3g08760 | 8670.3674 | 8605.9649 | 1.00748347 | DOWN | 1.00682513 | 1191 | 3 | 2241507 | 2242697 | - | tRNA m(1)G methyltransferase  domain containingprotein |
|  | 9661.3075 | 9602.0934 | 1.00616679 | DOWN |  |  |  |  |  |  |
| Afu3g09330 | 10432.368 | 10497.6745 | 1.00625999 | UP | 1.00605592 | 1680 | 3 | 2361848 | 2363527 | - | conserved hypothetical  protein |
|  | 10573.3662 | 10511.8525 | 1.00585184 | DOWN |  |  |  |  |  |  |
| Afu3g10110 | 12946.7339 | 12962.8981 | 1.00124852 | UP | 1.00701232 | 2436 | 3 | 2603190 | 2605625 | - | electron transfer flavoprotein-  ubiquinoneoxidoreductase |
|  | 13286.6416 | 13456.3933 | 1.01277612 | UP |  |  |  |  |  |  |
| Afu3g11080 | 9751.2271 | 9747.8412 | 1.00034735 | DOWN | 1.0049101 | 4980 | 3 | 2893788 | 2898767 | + | MAP kinase kinase kinase  (Bck1), putative |
|  | 8473.6342 | 8553.9037 | 1.00947285 | UP |  |  |  |  |  |  |
| Afu3g14270 | 2819.5379 | 2851.9729 | 1.01150366 | UP | 1.0068698 | 1136 | 3 | 3790469 | 3791604 | + | aldo-keto reductase (AKR),  putative |
|  | 2847.7947 | 2854.1622 | 1.00223594 | UP |  |  |  |  |  |  |
| Afu4g00540 | 4237.4598 | 4253.1286 | 1.00369769 | UP | 1.00534369 | 2094 | 4 | 150004 | 152097 | + | dynamin GTPase, putative |
|  | 3819.3794 | 3846.0757 | 1.0069897 | UP |  |  |  |  |  |  |
| Afu4g07390 | 10319.412 | 10294.616 | 1.00240864 | DOWN | 1.0070805 | 850 | 4 | 1916919 | 1917768 | + | endosomal cargo receptor  (Erp5), putative |
|  | 10428.7294 | 10551.2917 | 1.01175237 | UP |  |  |  |  |  |  |
| Afu4g08440 | 4769.2831 | 4783.5331 | 1.00298787 | UP | 1.00155072 | 2500 | 4 | 2175884 | 2178383 | - | Patatin-like serine hydrolase,  putative |
|  | 4911.743 | 4912.3008 | 1.00011356 | UP |  |  |  |  |  |  |
| Afu4g10590 | 3039.4065 | 3000.5331 | 1.0129555 | DOWN | 1.00663818 | 484 | 4 | 2766062 | 2766545 | - | hypothetical protein |
|  | 3031.5055 | 3030.5331 | 1.00032087 | DOWN |  |  |  |  |  |  |
| Afu4g12020 | 3113.5331 | 3119.0331 | 1.00176648 | UP | 1.00174183 | 2337 | 4 | 3157512 | 3159848 | + | hypothetical protein |
|  | 3198.6707 | 3204.1634 | 1.00171718 | UP |  |  |  |  |  |  |
| Afu4g13570 | 5274.1879 | 5223.3452 | 1.00973374 | DOWN | 1.00829794 | 978 | 4 | 3546096 | 3547073 | - | thiol methyltransferase 1 |
|  | 5164.206 | 5129.01 | 1.00686214 | DOWN |  |  |  |  |  |  |
| Afu4g13850 | 2797.7831 | 2836.4785 | 1.01383074 | UP | 1.00760691 | 1352 | 4 | 3612167 | 3613518 | - | conserved hypothetical  protein |
|  | 2693.5331 | 2697.2585 | 1.00138309 | UP |  |  |  |  |  |  |
| Afu5g01670 | 3931.1251 | 3915.6357 | 1.00395578 | DOWN | 1.00397099 | 1279 | 5 | 422538 | 423816 | + | hypothetical protein |
|  | 4155.7831 | 4139.2831 | 1.0039862 | DOWN |  |  |  |  |  |  |
| Afu5g11340 | 11117.5331 | 11090.5696 | 1.00243121 | DOWN | 1.00754518 | 987 | 5 | 2914909 | 2915895 | - | translation initiation factor  eIF-2B alphasubunit, putative |
|  | 11713.5331 | 11567.1034 | 1.01265915 | DOWN |  |  |  |  |  |  |
| Afu5g13600 | 6517.0152 | 6474.5227 | 1.00656303 | DOWN | 1.00479157 | 2890 | 5 | 3569046 | 3571935 | + | vacuolar protein sorting  vps16, putative |
|  | 6247.8525 | 6229.0401 | 1.00302011 | DOWN |  |  |  |  |  |  |
| Afu5g13680 | 3891.7831 | 3872.2831 | 1.00503579 | DOWN | 1.00395785 | 351 | 5 | 3591753 | 3592103 | - | hypothetical protein |
|  | 3571.6553 | 3561.3988 | 1.00287991 | DOWN |  |  |  |  |  |  |
| Afu5g15050 | 2570.4741 | 2579.9532 | 1.00368769 | UP | 1.00677398 | 585 | 5 | 3895482 | 3896066 | - | trichodiene oxygenase |
|  | 2578.4251 | 2603.8491 | 1.00986028 | UP |  |  |  |  |  |  |
| Afu5g15070 | 2246.9715 | 2246.5063 | 1.00020708 | DOWN | 1.00524125 | 636 | 5 | 3901221 | 3901856 | - | antioxidant protein LsfA |
|  | 2494.6453 | 2469.2725 | 1.01027542 | DOWN |  |  |  |  |  |  |
| Afu6g00140 | 4694.8025 | 4691.0735 | 1.00079491 | DOWN | 1.00256896 | 1668 | 6 | 41091 | 42758 | + | hypothetical protein |
|  | 4567.2831 | 4547.5331 | 1.00434301 | DOWN |  |  |  |  |  |  |
| Afu6g00250 | 4048.2284 | 4066.4801 | 1.00450856 | UP | 1.00824014 | 1095 | 6 | 75194 | 76288 | + | GPI anchored protein,  putative |
|  | 3783.6424 | 3828.9391 | 1.01197172 | UP |  |  |  |  |  |  |
| Afu6g03090 | 2411.3766 | 2410.07 | 1.00054214 | DOWN | 1.00844295 | 1564 | 6 | 653049 | 654612 | - | cytochrome P450 alkane  hydroxylase, putative |
|  | 2456.21 | 2496.3537 | 1.01634376 | UP |  |  |  |  |  |  |
| Afu6g04280 | 2941.5331 | 2948.2831 | 1.00229472 | UP | 1.00790542 | 1313 | 6 | 962988 | 964300 | - | hypothetical protein |
|  | 2837.3597 | 2875.7098 | 1.01351612 | UP |  |  |  |  |  |  |
| Afu6g04450 | 3363.0398 | 3365.192 | 1.00063996 | UP | 1.00204391 | 1482 | 6 | 1016716 | 1018197 | + | alpha-1,2-mannosyltransferase  (Mnn2), putative |
|  | 3288.4296 | 3277.1305 | 1.00344786 | DOWN |  |  |  |  |  |  |
| Afu6g05300 | 11949.5343 | 12059.7256 | 1.00922139 | UP | 1.00754008 | 1006 | 6 | 1270239 | 1271244 | - | CCAAT-binding factor complex  subunit HapE |
|  | 12267.8478 | 12339.7222 | 1.00585876 | UP |  |  |  |  |  |  |
| Afu6g10540 | 10813.3592 | 10824.2222 | 1.00100459 | UP | 1.00522835 | 1457 | 6 | 2609862 | 2611318 | - | conserved hypothetical  protein |
|  | 10169.69 | 10074.465 | 1.00945211 | DOWN |  |  |  |  |  |  |
| Afu6g11770 | 1622.8893 | 1614.6056 | 1.00513048 | DOWN | 1.00588426 | 1070 | 6 | 2926410 | 2927479 | + | hypothetical protein |
|  | 1469.6235 | 1459.9324 | 1.00663805 | DOWN |  |  |  |  |  |  |
| Afu6g11910 | 5008.8844 | 4980.8582 | 1.00562678 | DOWN | 1.00401672 | 2834 | 6 | 2979806 | 2982639 | - | glycosyl hydrolase family 3,  putative |
|  | 5102.2831 | 5090.0331 | 1.00240666 | DOWN |  |  |  |  |  |  |
| Afu6g13660 | 10976.8629 | 10794.5844 | 1.01688611 | DOWN | 1.00856218 | 4069 | 6 | 3478211 | 3482279 | - | conserved hypothetical  protein |
|  | 10087.8456 | 10085.4428 | 1.00023824 | DOWN |  |  |  |  |  |  |
| Afu6g13770 | 6224.3542 | 6246.8621 | 1.0036161 | UP | 1.00215457 | 2353 | 6 | 3518480 | 3520832 | + | C6 finger domain protein,  putative |
|  | 5790.6223 | 5786.612 | 1.00069303 | DOWN |  |  |  |  |  |  |
| Afu7g00470 | 5285.345 | 5281.4364 | 1.00074006 | DOWN | 1.00208083 | 1494 | 7 | 122689 | 124182 | - | hypothetical protein |
|  | 5332.0593 | 5313.8774 | 1.00342159 | DOWN |  |  |  |  |  |  |
| Afu7g01800 | 12012.0137 | 12124.3966 | 1.00935588 | UP | 1.00485875 | 2766 | 7 | 477654 | 480419 | + | AT DNA binding protein,  putative |
|  | 11965.1145 | 11960.7892 | 1.00036162 | DOWN |  |  |  |  |  |  |
| Afu7g06410 | 5191.3791 | 5207.414 | 1.00308876 | UP | 1.00533248 | 1724 | 7 | 1571892 | 1573615 | - | exo-polygalacturonase,  putative |
|  | 4809.3164 | 4845.7528 | 1.00757621 | UP |  |  |  |  |  |  |
