# Supplementary material for: Genome-Wide Maps of Mononucleosomes and Dinucleosomes Containing Hyperacetylated Histones of Aspergillus fumigatus
Source: PLoS One. 2010 Mar 26;5(3):e9916. doi: 10.1371/journal.pone.0009916 (PMC2845647; doi:10.1371/journal.pone.0009916)
Supplement: Table S5 — Expression levels of the genes with more than 2 fold changes between the TSA-treated and untreated cells. (0.37 MB DOC) [file pone.0009916.s005.doc]

| Supplementary Table S5. Expression levels of the genes with more than 2 fold changes between the TSA-treated and untreated cells. | | | | | | | | | | |
| --- | --- | --- | --- | --- | --- | --- | --- | --- | --- | --- |
| Gene | Intensity (w/o TSA) | Intensity (with TSA) | Fold changes | Down/Up | Length | Chromosome | Gene body | | Strand | Annotation |
| Afu1g00990 | 7513.2 | 3284.9 | 2.3 | DOWN | 881 | 1 | 347632 | 348512 | - | short chain dehydrogenase/reductase  familyprotein |
|  | 8326.4 | 3941.7 | 2.1 | DOWN |  |  |  |  |  |
| Afu1g03570 | 46982.6 | 8958.7 | 5.2 | DOWN | 1457 | 1 | 1031631 | 1033087 | + | acid phosphatase PHOa |
|  | 46349.8 | 11359.4 | 4.1 | DOWN |  |  |  |  |  |
| Afu1g04430 | 10731.3 | 4875.4 | 2.2 | DOWN | 1275 | 1 | 1258607 | 1259881 | + | hypothetical protein |
|  | 10565.1 | 5412.6 | 2.0 | DOWN |  |  |  |  |  |
| Afu1g04575 | 10534.7 | 4000.6 | 2.6 | DOWN | 912 | 1 | 1296944 | 1297855 | + | hypothetical protein |
|  | 9050.7 | 3760.3 | 2.4 | DOWN |  |  |  |  |  |
| Afu1g10930 | 13345.8 | 5493.9 | 2.4 | DOWN | 1584 | 1 | 2853321 | 2854904 | - | ammonium transporter |
|  | 13536.1 | 6507.8 | 2.1 | DOWN |  |  |  |  |  |
| Afu1g11500 | 17661.0 | 6081.1 | 2.9 | DOWN | 1242 | 1 | 3037340 | 3038581 | + | hypothetical protein |
|  | 15786.3 | 6161.3 | 2.6 | DOWN |  |  |  |  |  |
| Afu1g16030 | 7110.6 | 2582.8 | 2.8 | DOWN | 1391 | 1 | 4358866 | 4360256 | - | conserved hypothetical protein |
|  | 5836.8 | 2591.2 | 2.3 | DOWN |  |  |  |  |  |
| Afu1g17590 | 19127.0 | 3569.0 | 5.4 | DOWN | 1421 | 1 | 4823281 | 4824701 | + | phosphoesterase superfamily protein |
|  | 17546.3 | 3767.2 | 4.7 | DOWN |  |  |  |  |  |
| Afu2g02030 | 13742.2 | 3695.8 | 3.7 | DOWN | 1397 | 2 | 503811 | 505207 | - | fructosyl amine:oxygen  oxidoreductase |
|  | 12712.6 | 3732.0 | 3.4 | DOWN |  |  |  |  |  |
| Afu2g08340 | 10871.5 | 4664.0 | 2.3 | DOWN | 1248 | 2 | 2144609 | 2145856 | - | hypothetical protein |
|  | 10913.4 | 5138.7 | 2.1 | DOWN |  |  |  |  |  |
| Afu2g08820 | 14513.9 | 6097.3 | 2.4 | DOWN | 954 | 2 | 2267613 | 2268566 | + | hypothetical protein |
|  | 13154.0 | 6220.6 | 2.1 | DOWN |  |  |  |  |  |
| Afu2g10940 | 6890.4 | 2797.1 | 2.5 | DOWN | 555 | 2 | 2815982 | 2816536 | + | hypothetical protein |
|  | 7404.9 | 3191.9 | 2.3 | DOWN |  |  |  |  |  |
| Afu2g13500 | 21484.7 | 10155.2 | 2.1 | DOWN | 2961 | 2 | 3502024 | 3504984 | - | hypothetical protein |
|  | 21045.9 | 10048.8 | 2.1 | DOWN |  |  |  |  |  |
| Afu3g06020 | 19507.3 | 6951.0 | 2.8 | DOWN | 1782 | 3 | 1479927 | 1481708 | + | glyoxalase family protein family |
|  | 20943.7 | 7657.0 | 2.7 | DOWN |  |  |  |  |  |
| Afu3g06720 | 9791.9 | 3646.6 | 2.7 | DOWN | 1087 | 3 | 1667616 | 1668702 | - | ThiJ/PfpI family protein |
|  | 10692.1 | 4311.9 | 2.5 | DOWN |  |  |  |  |  |
| Afu3g13670 | 7711.3 | 2770.0 | 2.8 | DOWN | 1843 | 3 | 3605885 | 3607727 | + | siderochrome-iron transporter, putative |
|  | 7288.5 | 2796.0 | 2.6 | DOWN |  |  |  |  |  |
| Afu4g03240 | 4925.4 | 2065.4 | 2.4 | DOWN | 855 | 4 | 911258 | 912112 | + | cell wall galactomannoprotein Mp1 |
|  | 5155.6 | 2313.0 | 2.2 | DOWN |  |  |  |  |  |
| Afu4g03660 | 35447.0 | 7805.7 | 4.5 | DOWN | 1307 | 4 | 1028054 | 1029360 | + | acid phosphatase, putative |
|  | 32873.2 | 7398.7 | 4.4 | DOWN |  |  |  |  |  |
| Afu4g08370 | 15887.6 | 6558.1 | 2.4 | DOWN | 1260 | 4 | 2162562 | 2163821 | - | conserved hypothetical protein |
|  | 15652.8 | 6741.3 | 2.3 | DOWN |  |  |  |  |  |
| Afu4g08380 | 8520.4 | 3493.1 | 2.4 | DOWN | 807 | 4 | 2165277 | 2166083 | - | hypothetical protein |
|  | 7636.1 | 3526.2 | 2.2 | DOWN |  |  |  |  |  |
| Afu4g08630 | 13406.1 | 3135.1 | 4.3 | DOWN | 1455 | 4 | 2237969 | 2239423 | + | phytase |
|  | 11097.5 | 3922.4 | 2.8 | DOWN |  |  |  |  |  |
| Afu4g12700 | 18132.8 | 5651.8 | 3.2 | DOWN | 1110 | 4 | 3328979 | 3330088 | - | hypothetical protein |
|  | 17306.1 | 6020.8 | 2.9 | DOWN |  |  |  |  |  |
| Afu4g12870 | 17656.9 | 6923.9 | 2.6 | DOWN | 1943 | 4 | 3364936 | 3366878 | + | methylmalonate-semialdehyde  dehydrogenase,putative |
|  | 17170.0 | 8088.7 | 2.1 | DOWN |  |  |  |  |  |
| Afu5g00730 | 14717.3 | 5989.0 | 2.5 | DOWN | 3585 | 5 | 205171 | 208755 | - | H /K ATPase alpha subunit, putative |
|  | 13321.9 | 5875.0 | 2.3 | DOWN |  |  |  |  |  |
| Afu5g02820 | 5349.5 | 2357.7 | 2.3 | DOWN | 667 | 5 | 749896 | 750562 | - | hypothetical protein |
|  | 5344.3 | 2383.8 | 2.2 | DOWN |  |  |  |  |  |
| Afu5g03540 | 6716.7 | 2776.8 | 2.4 | DOWN | 1287 | 5 | 954371 | 955657 | + | pyridine nucleotide-disulphide  oxidoreductase,class II, putative |
|  | 6858.5 | 3019.1 | 2.3 | DOWN |  |  |  |  |  |
| Afu5g07360 | 10090.0 | 4493.7 | 2.2 | DOWN | 2162 | 5 | 1839248 | 1841409 | - | peroxisomal copper amine oxidase |
|  | 9957.9 | 4651.4 | 2.1 | DOWN |  |  |  |  |  |
| Afu5g08800 | 25299.2 | 10099.6 | 2.5 | DOWN | 857 | 5 | 2256072 | 2256928 | + | hypothetical protein |
|  | 24666.5 | 10624.6 | 2.3 | DOWN |  |  |  |  |  |
| Afu5g08940 | 5354.0 | 2654.1 | 2.0 | DOWN | 1809 | 5 | 2294281 | 2296089 | + | 3-methylcrotonyl-CoA carboxylase,  beta subunit,putative |
|  | 5351.1 | 2718.2 | 2.0 | DOWN |  |  |  |  |  |
| Afu5g09120 | 6967.8 | 3135.0 | 2.2 | DOWN | 1573 | 5 | 2343530 | 2345102 | - | neutral amino acid permease, putative |
|  | 6596.7 | 3242.2 | 2.0 | DOWN |  |  |  |  |  |
| Afu5g09130 | 8941.0 | 3847.4 | 2.3 | DOWN | 1040 | 5 | 2345689 | 2346728 | - | polysaccharide deacetylase family  protein |
|  | 8346.6 | 3897.8 | 2.1 | DOWN |  |  |  |  |  |
| Afu5g09140 | 17749.4 | 7137.7 | 2.5 | DOWN | 1713 | 5 | 2347320 | 2349032 | + | amidase |
|  | 16278.4 | 7029.0 | 2.3 | DOWN |  |  |  |  |  |
| Afu5g09150 | 11779.5 | 5225.7 | 2.3 | DOWN | 671 | 5 | 2349121 | 2349791 | - | oxidoreductase, short-chaindehydrogenase  /reductase family |
|  | 11573.8 | 5637.1 | 2.1 | DOWN |  |  |  |  |  |
| Afu5g09720 | 9345.8 | 4540.1 | 2.1 | DOWN | 1682 | 5 | 2514367 | 2516048 | - | flavin containing polyamine oxidase,  putative |
|  | 9401.8 | 4809.8 | 2.0 | DOWN |  |  |  |  |  |
| Afu5g10520 | 9089.7 | 3870.6 | 2.3 | DOWN | 2647 | 5 | 2688350 | 2690996 | + | alpha-1,2-mannosidase family protein |
|  | 9194.1 | 4359.0 | 2.1 | DOWN |  |  |  |  |  |
| Afu5g13250 | 8086.5 | 3659.9 | 2.2 | DOWN | 1058 | 5 | 3486057 | 3487114 | + | DUF614 domain protein |
|  | 7391.0 | 3656.5 | 2.0 | DOWN |  |  |  |  |  |
| Afu5g14890 | 11119.9 | 4589.3 | 2.4 | DOWN | 399 | 5 | 3850278 | 3850676 | - | hypothetical protein |
|  | 11887.8 | 5002.0 | 2.4 | DOWN |  |  |  |  |  |
| Afu5g14930 | 5128.7 | 2237.2 | 2.3 | DOWN | 1304 | 5 | 3863218 | 3864521 | - | conserved hypothetical protein |
|  | 5009.7 | 2288.6 | 2.2 | DOWN |  |  |  |  |  |
| Afu6g07370 | 15059.7 | 6967.0 | 2.2 | DOWN | 1619 | 6 | 1661321 | 1662939 | + | hypothetical protein |
|  | 15647.2 | 7582.0 | 2.1 | DOWN |  |  |  |  |  |
| Afu6g08760 | 16002.5 | 7166.0 | 2.2 | DOWN | 1505 | 6 | 2076082 | 2077586 | + | proline oxidase |
|  | 14901.1 | 6992.2 | 2.1 | DOWN |  |  |  |  |  |
| Afu6g10040 | 9169.5 | 4442.3 | 2.1 | DOWN | 1378 | 6 | 2470461 | 2471838 | - | fructosyl amine: oxygen oxidoreductase |
|  | 8002.3 | 3944.8 | 2.0 | DOWN |  |  |  |  |  |
| Afu6g12240 | 7370.1 | 2005.5 | 3.7 | DOWN | 1326 | 6 | 3075736 | 3077061 | - | Glycerophosphoryl diester  phosphodiesterasefamily family |
|  | 7278.1 | 2298.9 | 3.2 | DOWN |  |  |  |  |  |
| Afu7g03900 | 19767.5 | 7698.5 | 2.6 | DOWN | 1607 | 7 | 873236 | 874842 | - | hypothetical protein |
|  | 18457.6 | 7285.6 | 2.5 | DOWN |  |  |  |  |  |
| Afu7g04570 | 15429.0 | 7633.2 | 2.0 | DOWN | 3318 | 7 | 1039096 | 1042413 | + | Na/K ATPase alpha 1 subunit, putative |
|  | 13962.0 | 7034.9 | 2.0 | DOWN |  |  |  |  |  |
| Afu7g04910 | 24519.3 | 2937.0 | 8.3 | DOWN | 1791 | 7 | 1153683 | 1155473 | + | phosphatidylglycerol specific  phospholipase C,putative |
|  | 19800.0 | 3579.3 | 5.5 | DOWN |  |  |  |  |  |
| Afu7g06030 | 17096.2 | 5825.0 | 2.9 | DOWN | 1173 | 7 | 1477973 | 1479145 | - | alpha-ketoglutarate-dependent  taurinedioxygenase |
|  | 16501.0 | 5931.2 | 2.8 | DOWN |  |  |  |  |  |
| Afu7g06350 | 20160.2 | 6961.8 | 2.9 | DOWN | 2083 | 7 | 1555681 | 1557763 | - | phosphate transporter, putative |
|  | 20062.4 | 7515.5 | 2.7 | DOWN |  |  |  |  |  |
| Afu8g04310 | 6521.9 | 2671.9 | 2.4 | DOWN | 3726 | 8 | 964858 | 968583 | - | conserved hypothetical protein |
|  | 6263.3 | 2748.9 | 2.3 | DOWN |  |  |  |  |  |
| Afu8g04620 | 23406.2 | 8413.2 | 2.8 | DOWN | 1581 | 8 | 1052510 | 1054090 | + | hypothetical protein |
|  | 22370.0 | 8148.1 | 2.7 | DOWN |  |  |  |  |  |
| Afu1g05070 | 4812.9 | 10753.2 | 2.2 | UP | 1617 | 1 | 1452042 | 1453658 | - | tRNA methyltransferase, putative |
|  | 4552.5 | 9306.3 | 2.0 | UP |  |  |  |  |  |
| Afu1g06670 | 3970.0 | 11782.6 | 3.0 | UP | 1853 | 1 | 1909097 | 1910949 | - | hypothetical protein |
|  | 4300.6 | 11851.0 | 2.8 | UP |  |  |  |  |  |
| Afu1g14450 | 2783.1 | 9038.9 | 3.2 | UP | 2927 | 1 | 3859781 | 3862707 | - | exo-beta-1,3-glucanase Exg0 |
|  | 3261.7 | 8238.2 | 2.5 | UP |  |  |  |  |  |
| Afu1g15000 | 13990.9 | 29968.2 | 2.1 | UP | 2147 | 1 | 4029547 | 4031693 | + | 2-isopropylmalate synthase |
|  | 14434.0 | 28222.4 | 2.0 | UP |  |  |  |  |  |
| Afu1g17630 | 6585.0 | 14013.5 | 2.1 | UP | 2334 | 1 | 4830612 | 4832945 | - | FAD/FMN-containing protein |
|  | 6491.0 | 12808.2 | 2.0 | UP |  |  |  |  |  |
| Afu2g00500 | 4658.3 | 11312.5 | 2.4 | UP | 1417 | 2 | 108274 | 109690 | - | conserved hypothetical protein |
|  | 5126.4 | 11893.0 | 2.3 | UP |  |  |  |  |  |
| Afu3g00710 | 4082.0 | 25134.2 | 6.2 | UP | 1074 | 3 | 160364 | 161437 | + | allergen, putative |
|  | 4742.2 | 26244.8 | 5.5 | UP |  |  |  |  |  |
| Afu3g01400 | 5010.2 | 10686.1 | 2.1 | UP | 4658 | 3 | 347964 | 352621 | + | ABC multidrug transporter, putative |
|  | 5342.9 | 10799.2 | 2.0 | UP |  |  |  |  |  |
| Afu3g08990 | 11357.5 | 24271.3 | 2.1 | UP | 1414 | 3 | 2297135 | 2298548 | - | hypothetical protein |
|  | 11474.1 | 23774.4 | 2.1 | UP |  |  |  |  |  |
| Afu3g09220 | 3684.0 | 10585.5 | 2.9 | UP | 3577 | 3 | 2337796 | 2341372 | - | fatty acid hydroxylase, putative |
|  | 3884.4 | 7919.0 | 2.0 | UP |  |  |  |  |  |
| Afu3g15080 | 2483.4 | 8159.9 | 3.3 | UP | 1649 | 3 | 3975051 | 3976699 | + | hypothetical protein |
|  | 2444.0 | 7604.7 | 3.1 | UP |  |  |  |  |  |
| Afu4g02810 | 6018.4 | 16726.3 | 2.8 | UP | 1005 | 4 | 786297 | 787301 | - | oxidoreductase, putative |
|  | 6115.8 | 13893.8 | 2.3 | UP |  |  |  |  |  |
| Afu4g04380 | 7638.4 | 17598.7 | 2.3 | UP | 963 | 4 | 1238344 | 1239306 | - | phenazine biosynthesis protein,  PhzF family |
|  | 7656.2 | 17576.1 | 2.3 | UP |  |  |  |  |  |
| Afu4g14380 | 3472.1 | 10021.5 | 2.9 | UP | 875 | 4 | 3798852 | 3799726 | - | conserved hypothetical protein |
|  | 3565.8 | 7904.0 | 2.2 | UP |  |  |  |  |  |
| Afu5g00120 | 4775.9 | 14143.1 | 3.0 | UP | 1441 | 5 | 5839 | 7279 | + | cytochrome P450 alkane hydroxylase,  putative |
|  | 5412.0 | 13947.0 | 2.6 | UP |  |  |  |  |  |
| Afu5g00920 | 2487.0 | 13173.2 | 5.3 | UP | 1227 | 5 | 241535 | 242761 | + | transesterase (LovD), putative |
|  | 2483.5 | 11268.3 | 4.5 | UP |  |  |  |  |  |
| Afu5g10120 | 5218.5 | 12855.5 | 2.5 | UP | 3825 | 5 | 2603086 | 2606910 | + | nonribosomal peptide synthase  (NRPS), putative |
|  | 5711.7 | 11625.8 | 2.0 | UP |  |  |  |  |  |
| Afu6g00290 | 8658.4 | 18311.9 | 2.1 | UP | 1111 | 6 | 84596 | 85706 | + | aminotransferase, putative |
|  | 9226.2 | 18807.8 | 2.0 | UP |  |  |  |  |  |
| Afu6g09740 | 4352.8 | 12630.5 | 2.9 | UP | 1063 | 6 | 2371240 | 2372302 | + | pyridine nucleotide-disulphide  oxidoreductase,putative |
|  | 4405.3 | 11040.9 | 2.5 | UP |  |  |  |  |  |
| Afu7g00120 | 5653.7 | 16234.3 | 2.9 | UP | 1041 | 7 | 27793 | 28833 | - | metallo-beta-lactamase domain  protein, putative |
|  | 5244.7 | 14137.4 | 2.7 | UP |  |  |  |  |  |
| Afu7g00130 | 3048.4 | 8040.0 | 2.6 | UP | 2472 | 7 | 29421 | 31892 | - | C6 transcription factor, putative |
|  | 3401.1 | 8737.5 | 2.6 | UP |  |  |  |  |  |
| Afu7g00150 | 4102.0 | 9675.8 | 2.4 | UP | 1280 | 7 | 33689 | 34968 | - | monooxygenase, putative |
|  | 3853.4 | 9064.3 | 2.4 | UP |  |  |  |  |  |
| Afu7g00160 | 3224.0 | 23943.2 | 7.4 | UP | 5573 | 7 | 36017 | 41589 | - | polyketide synthase type 1 (PKS),  putative |
|  | 3477.3 | 18748.3 | 5.4 | UP |  |  |  |  |  |
| Afu7g00170 | 4585.9 | 38799.6 | 8.5 | UP | 1362 | 7 | 42071 | 43432 | + | dimethylallyl tryptophan  synthase-related |
|  | 5170.7 | 38056.1 | 7.4 | UP |  |  |  |  |  |
| Afu7g00180 | 4707.8 | 15134.0 | 3.2 | UP | 948 | 7 | 44029 | 44976 | - | nucleoside-diphosphate sugar  epimerase,putative |
|  | 5442.1 | 17055.6 | 3.1 | UP |  |  |  |  |  |
| Afu7g00200 | 3082.8 | 13986.6 | 4.5 | UP | 1182 | 7 | 49143 | 50324 | + | hypothetical protein |
|  | 3244.9 | 13966.5 | 4.3 | UP |  |  |  |  |  |
| Afu7g04930 | 3773.9 | 7980.4 | 2.1 | UP | 1412 | 7 | 1161298 | 1162709 | + | alkaline serine protease (PR1),  putative |
|  | 3524.4 | 7081.3 | 2.0 | UP |  |  |  |  |  |
| Afu8g00610 | 5498.9 | 20755.0 | 3.8 | UP | 1151 | 8 | 143172 | 144322 | - | cell surface protein (Mas1),  putative |
|  | 5758.8 | 14723.8 | 2.6 | UP |  |  |  |  |  |
