# Supplementary figures and images for: Genome-Wide Maps of Mononucleosomes and Dinucleosomes Containing Hyperacetylated Histones of Aspergillus fumigatus
Source: PLoS One. 2010 Mar 26;5(3):e9916. doi: 10.1371/journal.pone.0009916 (PMC2845647; doi:10.1371/journal.pone.0009916)

## Slide 1
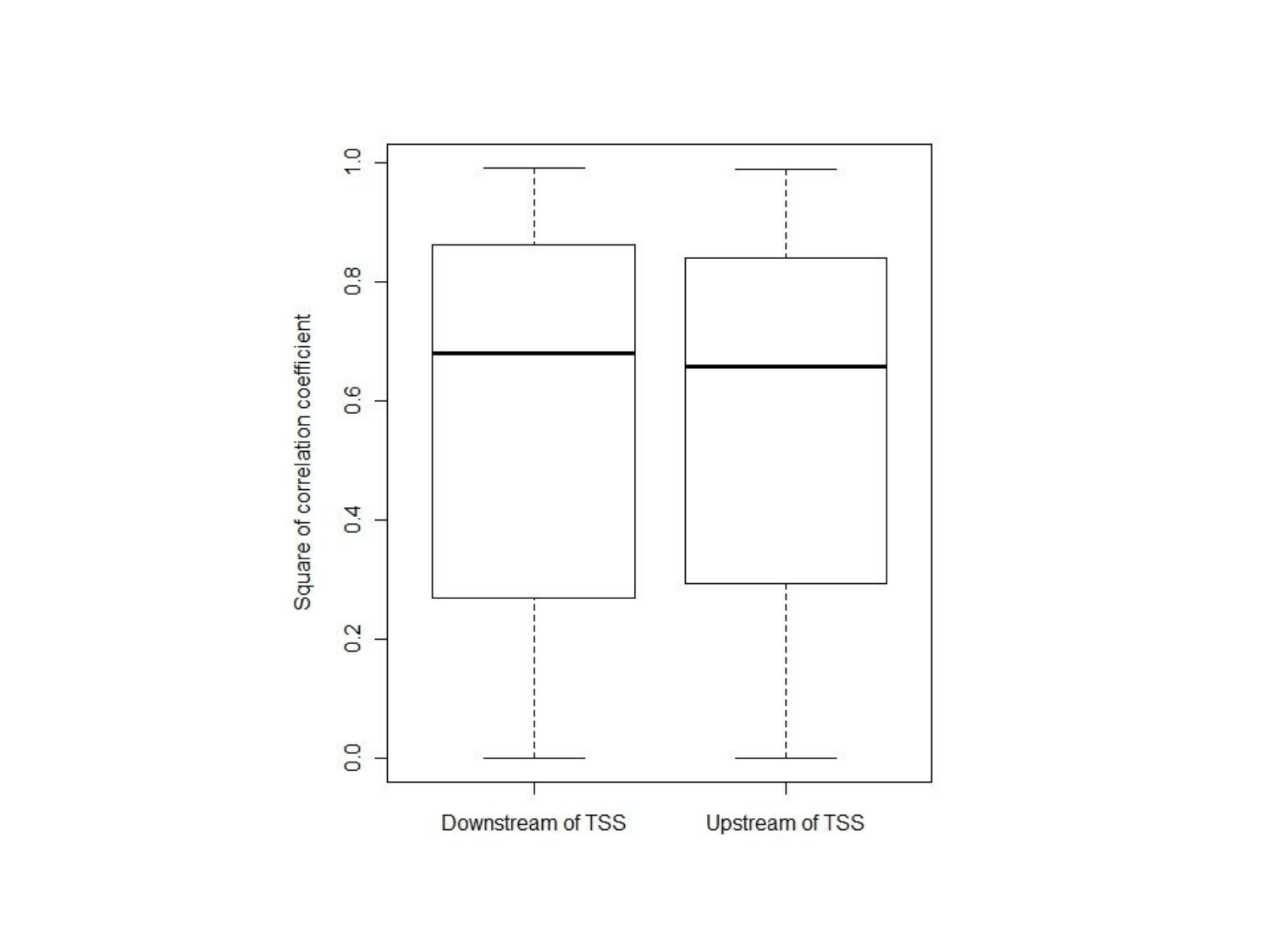

Supplement: Figure S1 — Boxplots of the squares of Pearson's correlation coefficient between the profiles of the TSA-treated (15-min treatment with MNase) and untreated mononucleosome mapping numbers in the 300 nt downstream and upstream of each of the 557 transcription start sites (>1000 sequence tags). (0.10 MB PPT) [file pone.0009916.s006.ppt]

## Slide 1
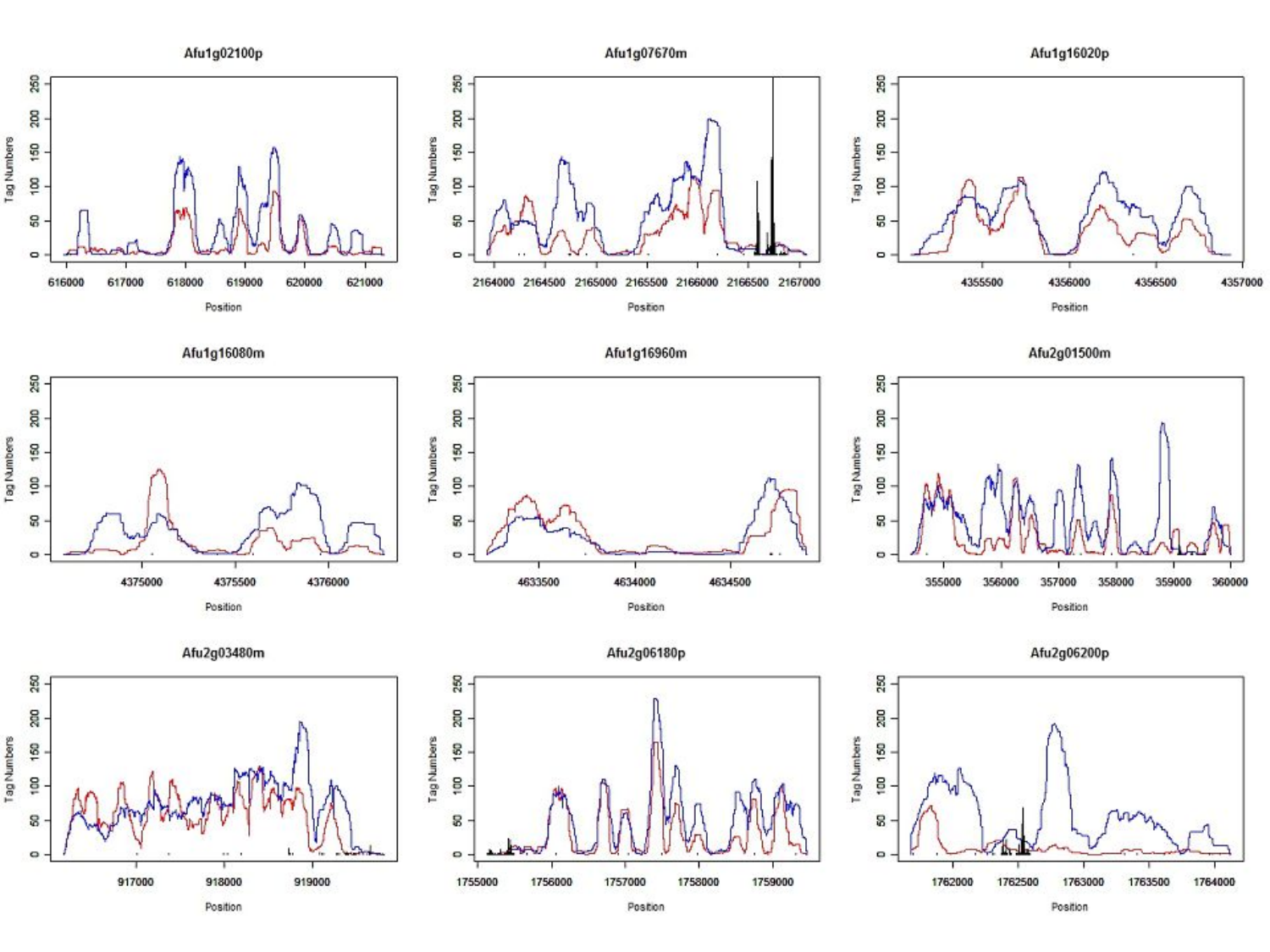

## Slide 2
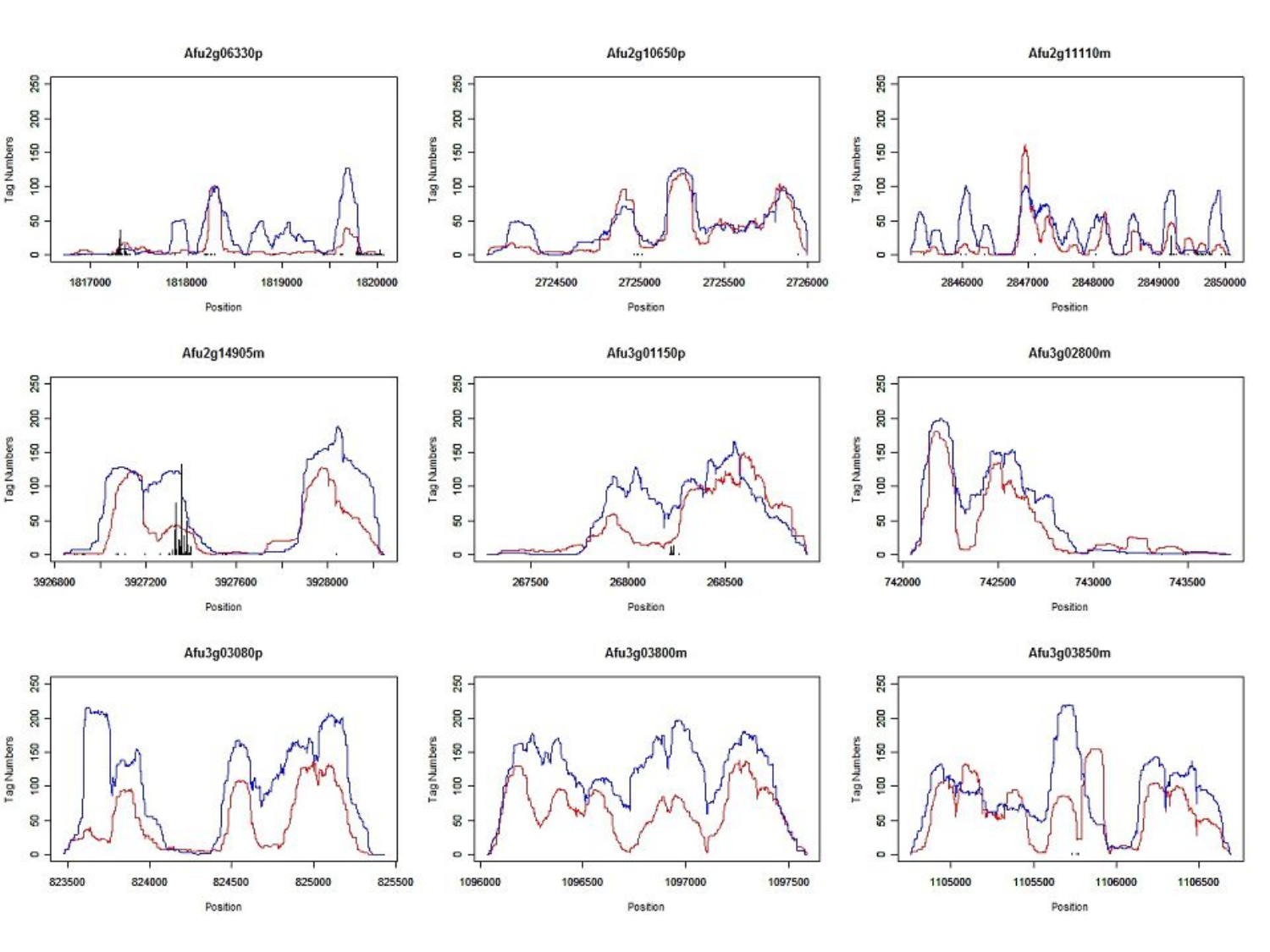

## Slide 3
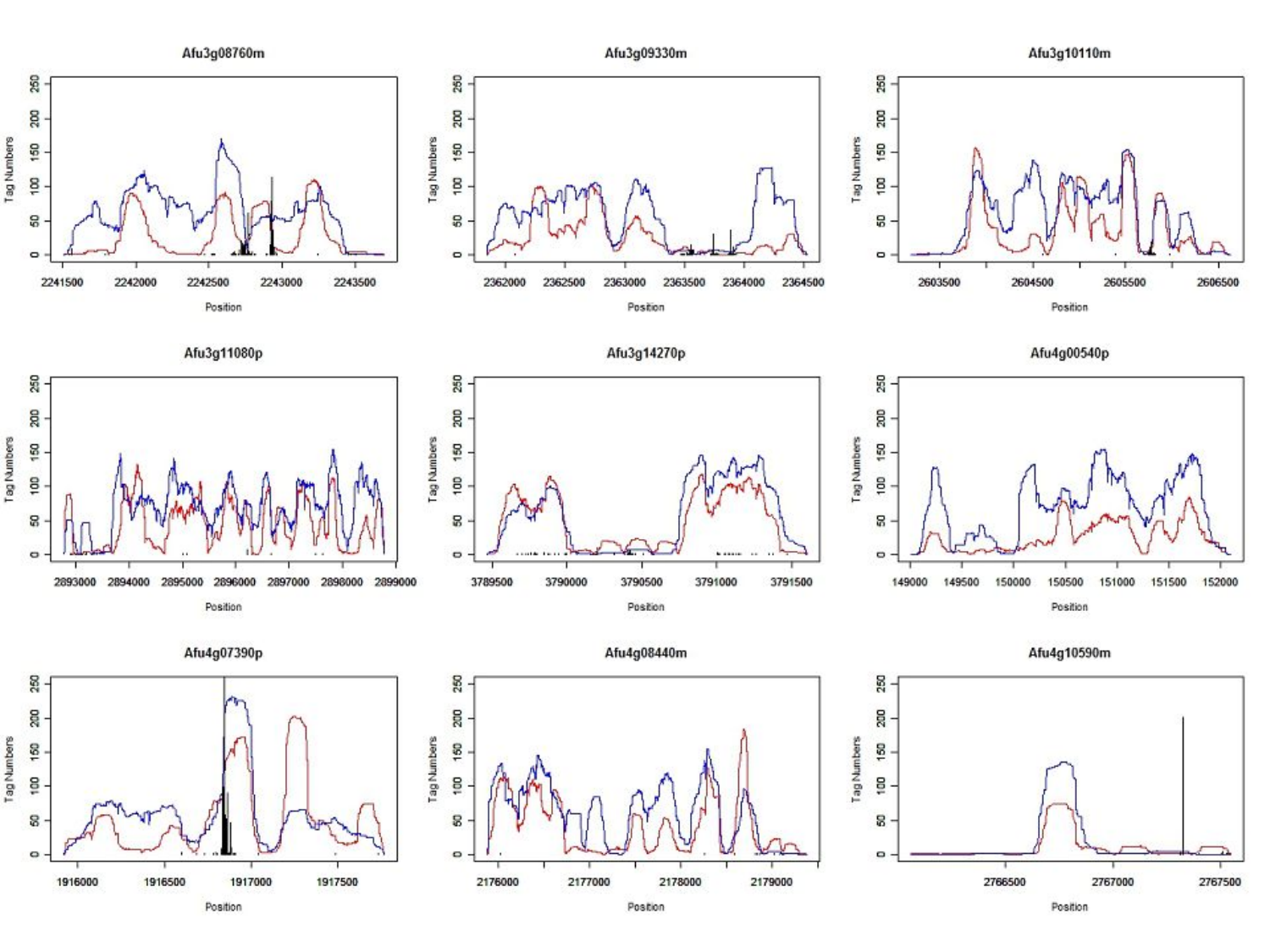

## Slide 4
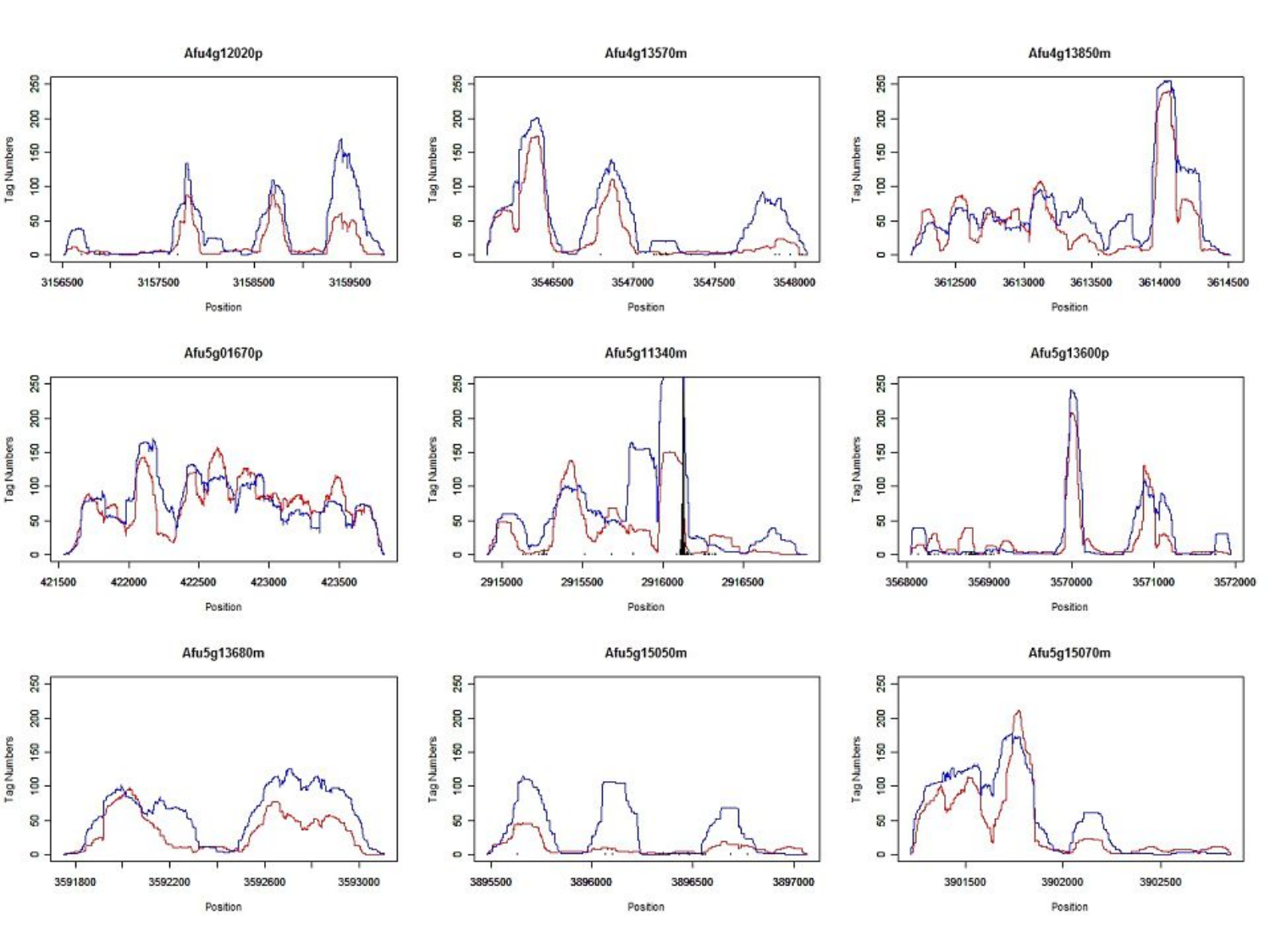

## Slide 5
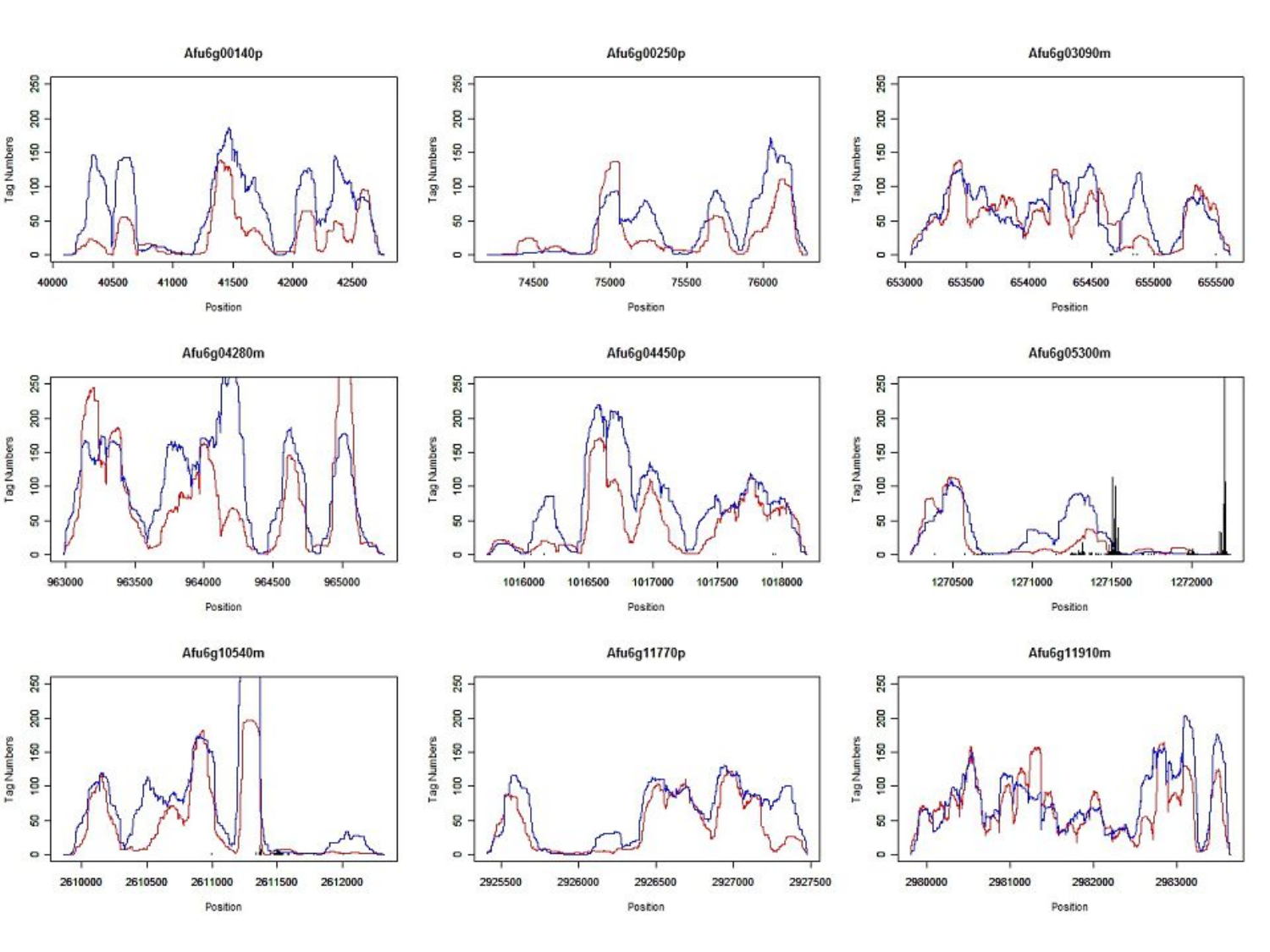

## Slide 6
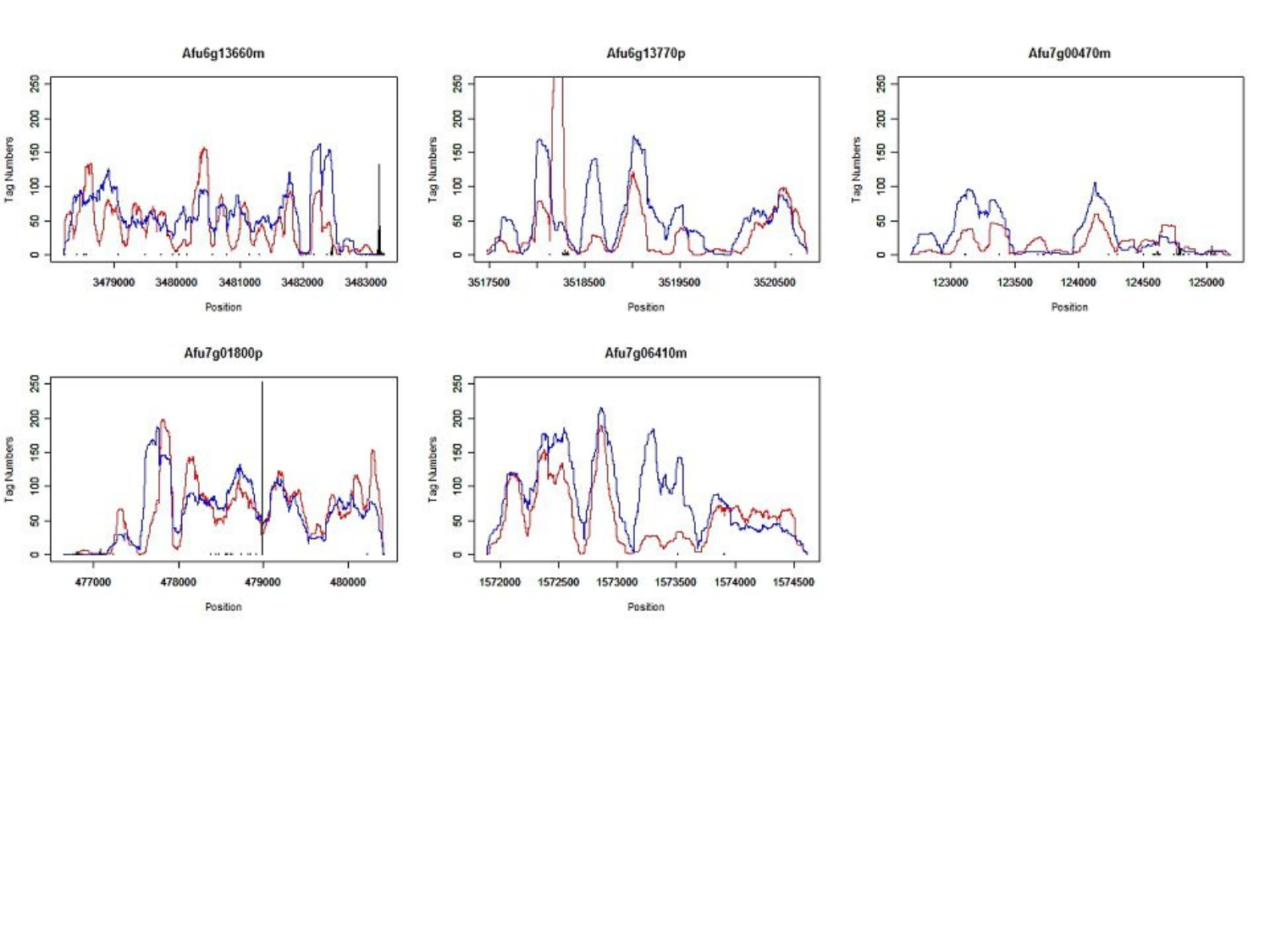

Supplement: Figure S2 — Mapping numbers of mononucleosomes and transcription start sites of the 50 constant expressed genes between the TSA-treated and untreated cells. Title indicates gene name with the last character “p” or “m”. The “p” indicates that the region between the positions 1 and 1,000 is the promoter and the other region is gene body. The “m” indicates that the region between the position 1,000 downstream from the last position and the last position is the promoter and the other region is gene body. Red and blue indicate the mononucleosome mapping number of the untreated cells and that of the TSA-treated cells respectively. The arrow indicates the region from the translational start to the end. The bars indicate the transcription start sites and the mapping numbers. (1.76 MB PPT) [file pone.0009916.s007.ppt]

## Slide 1
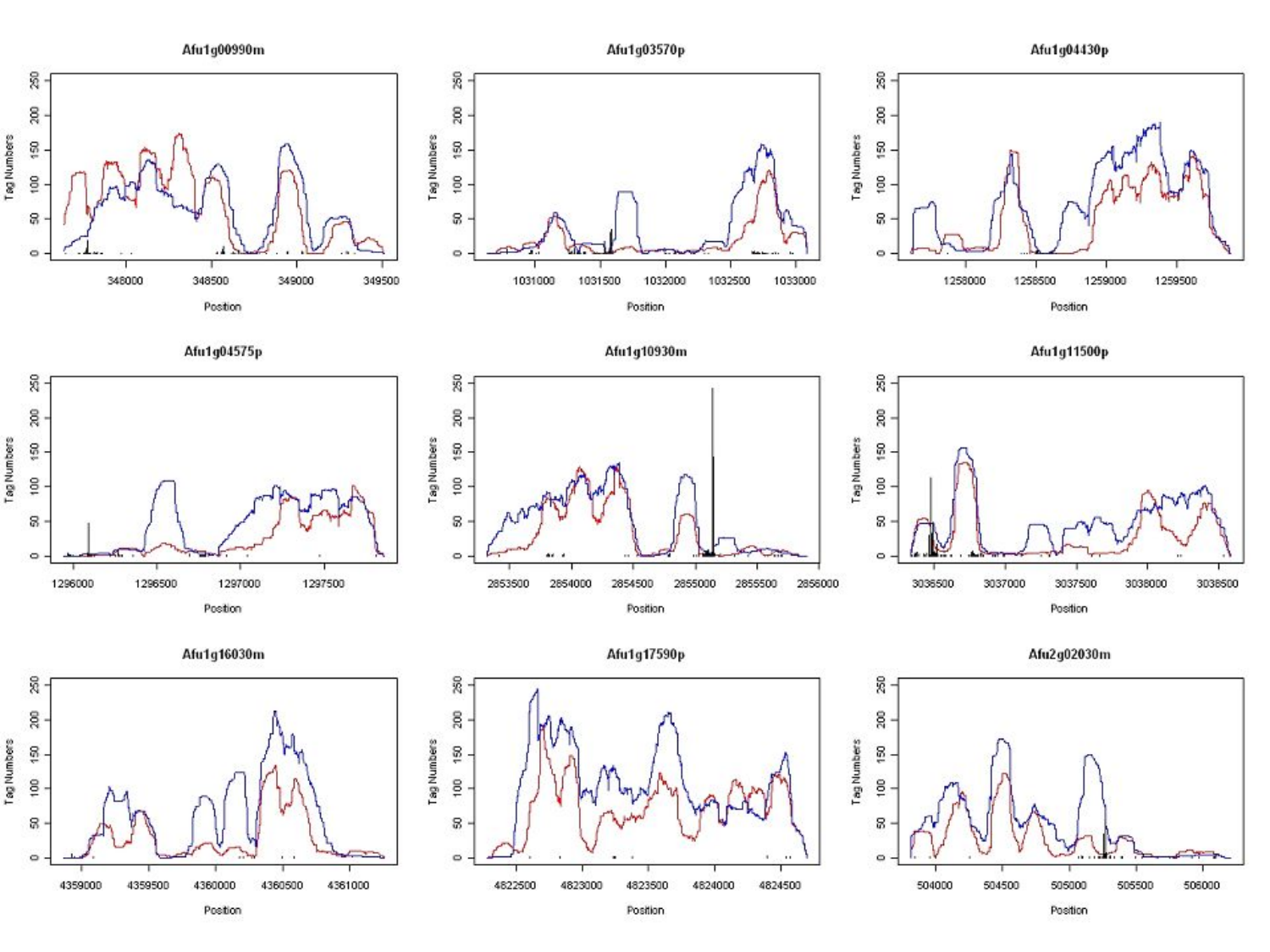

## Slide 2
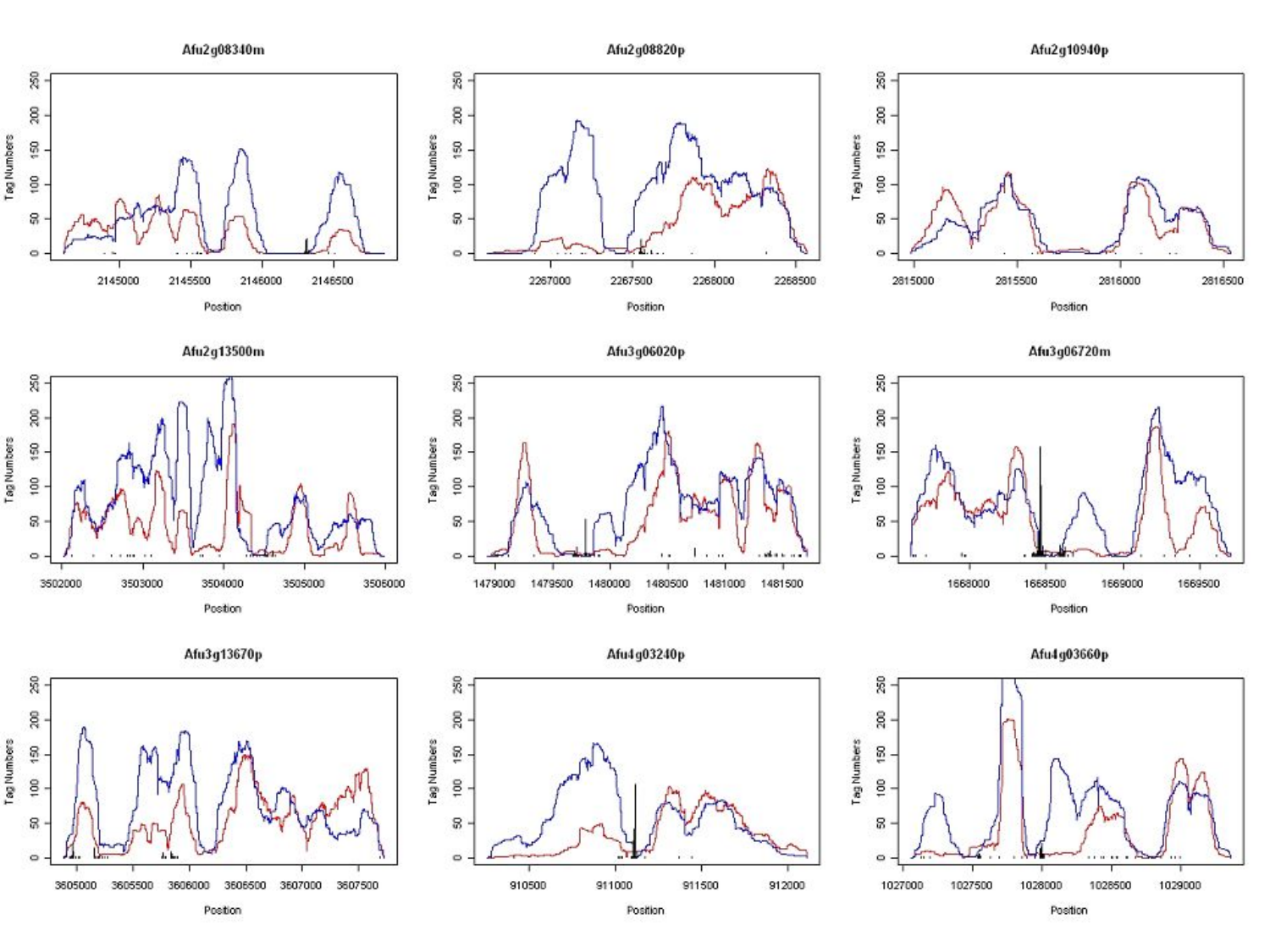

## Slide 3
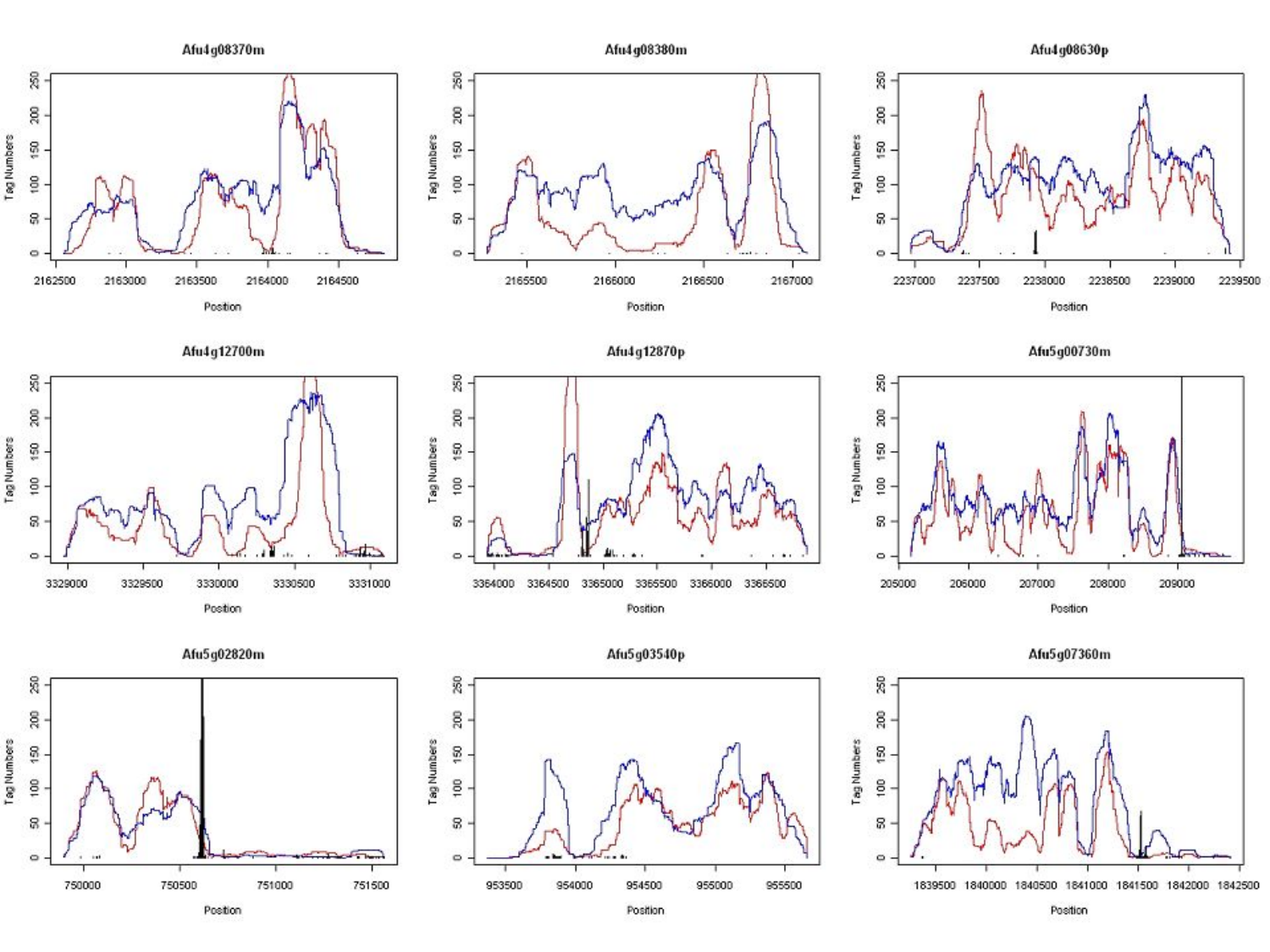

## Slide 4
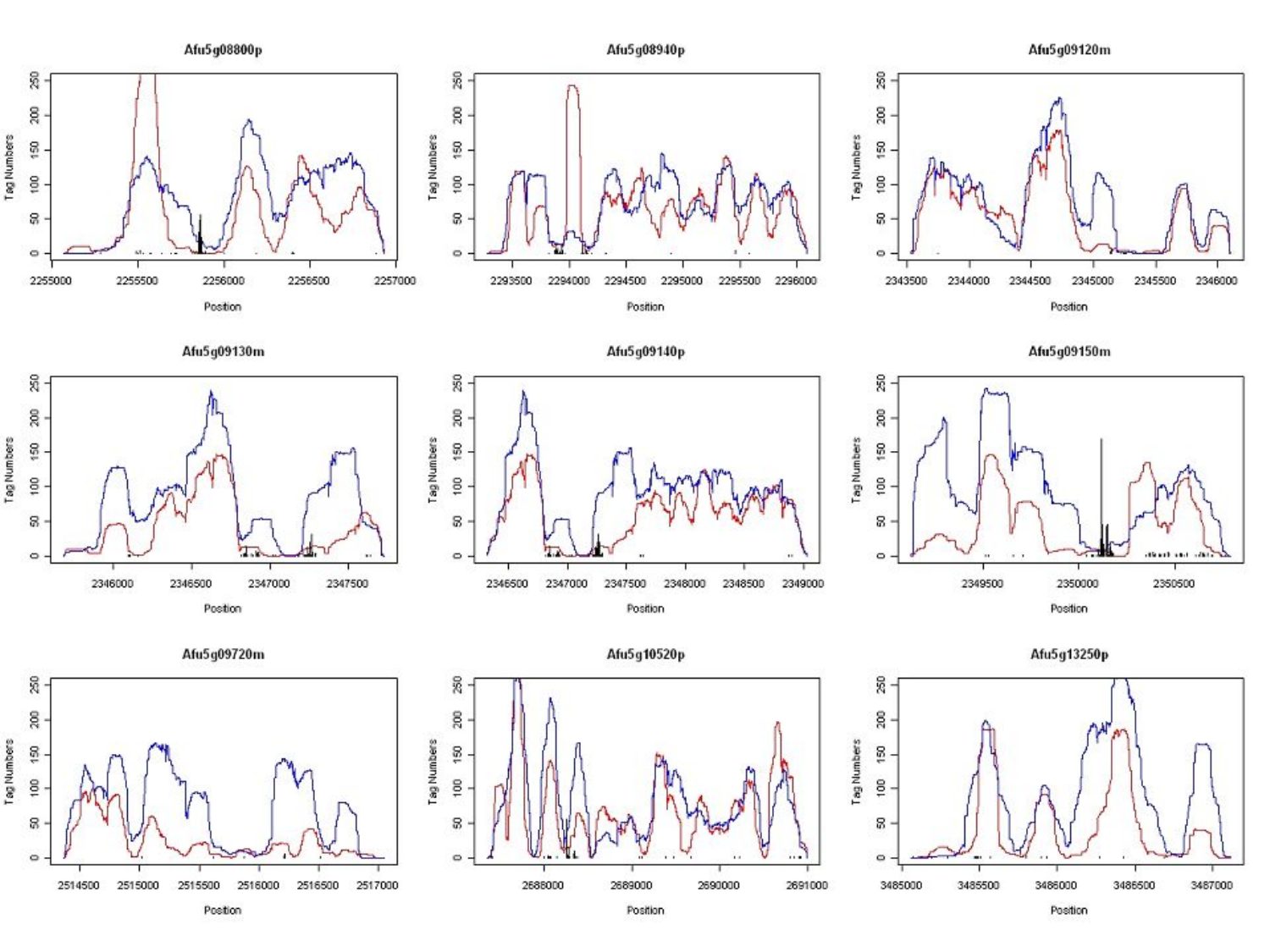

## Slide 5
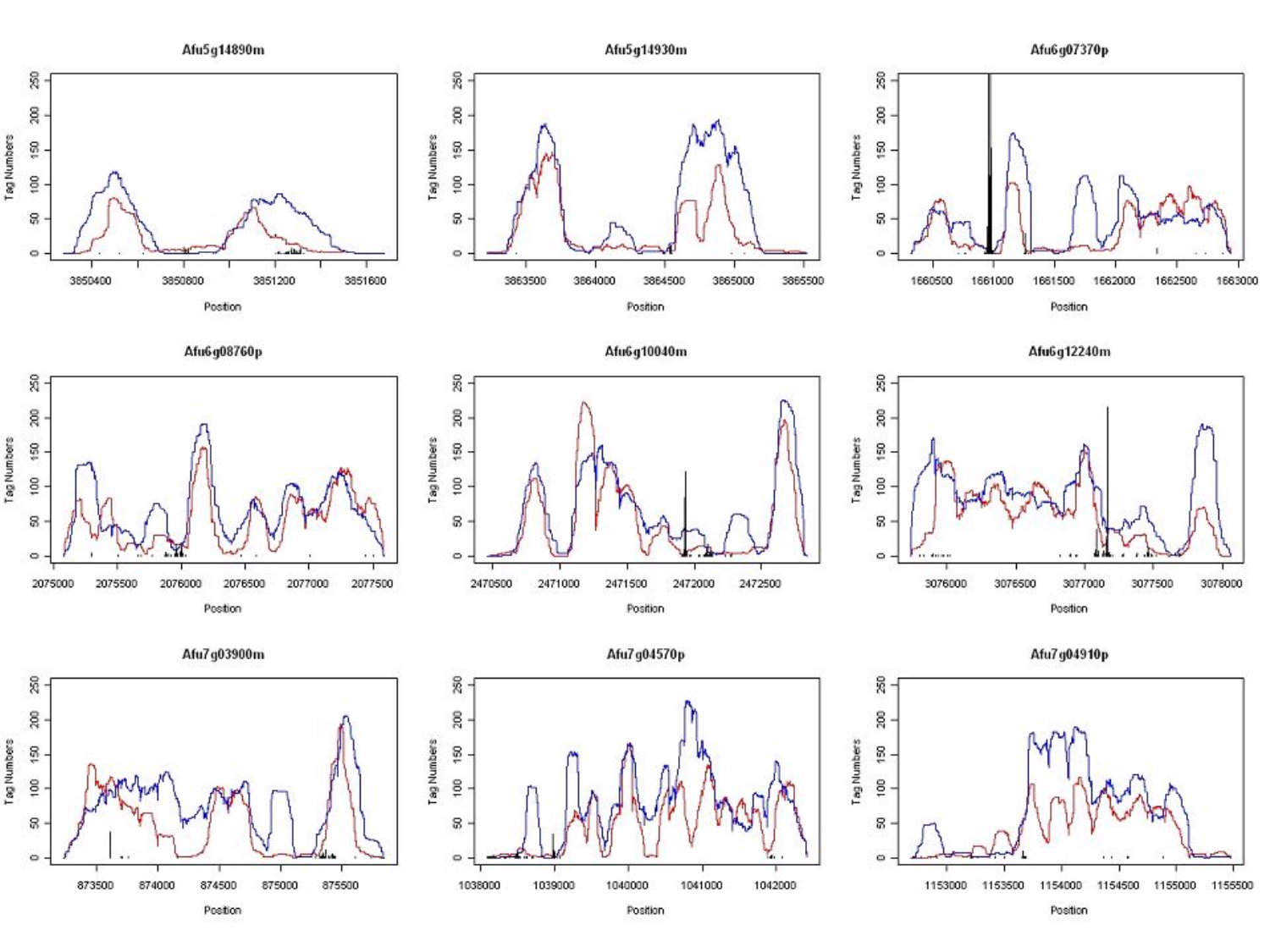

## Slide 6
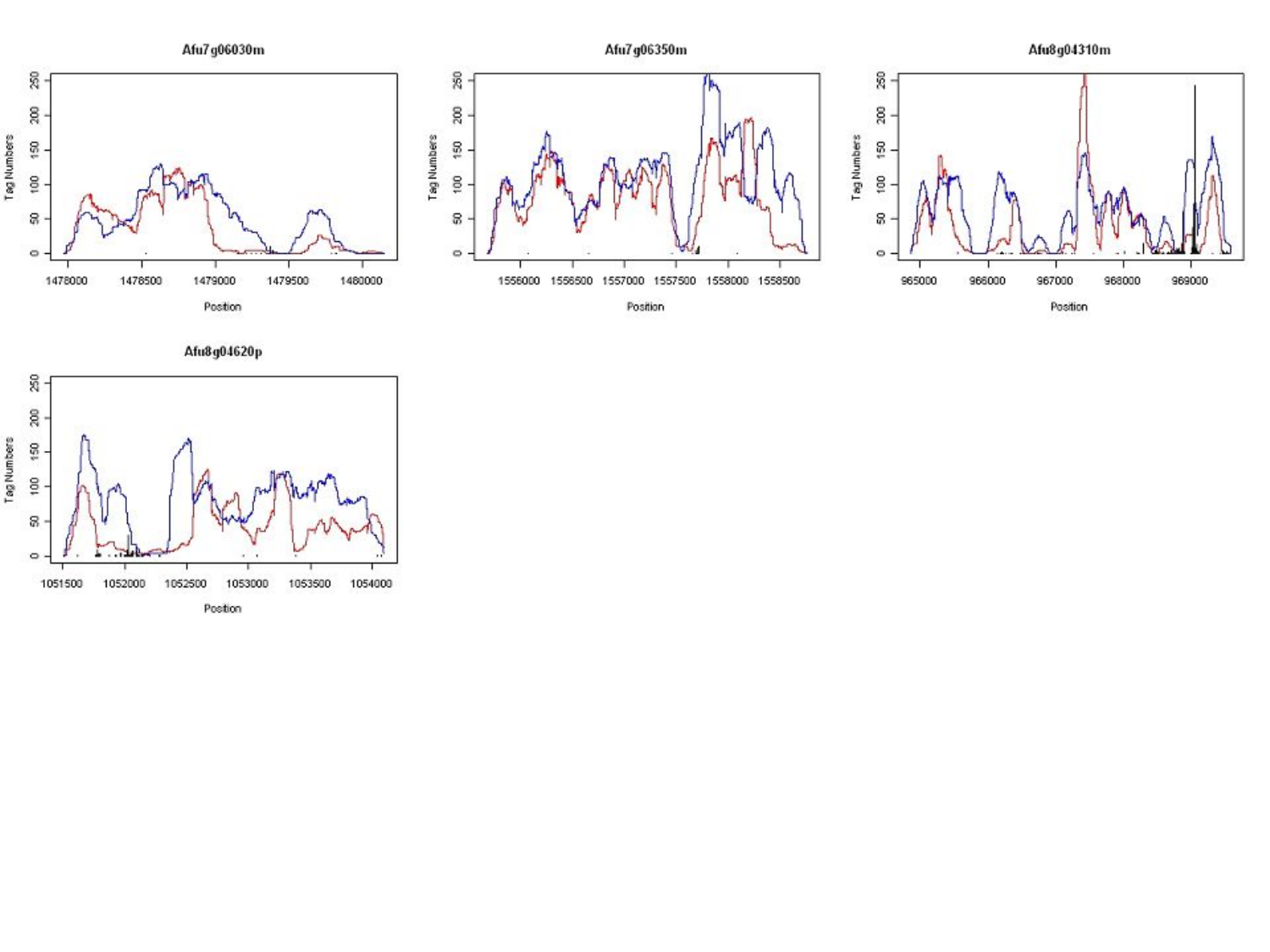

Supplement: Figure S3 — Mapping numbers of mononucleosomes and transcription start sites of the 49 down-regulated genes between the TSA-treated and untreated cells. Title indicates gene name with the last character “p” or “m”. The “p” indicates that the region between the positions 1 and 1,000 is the promoter and the other region is gene body. The “m” indicates that the region between the position 1,000 downstream from the last position and the last position is the promoter and the other region is gene body. Red and blue indicate the mononucleosome mapping number of the untreated cells and that of the TSA-treated cells respectively. The arrow indicates the region from the translational start to the end. The bars indicate the transcription start sites and the mapping numbers. (1.11 MB PPT) [file pone.0009916.s008.ppt]

## Slide 1
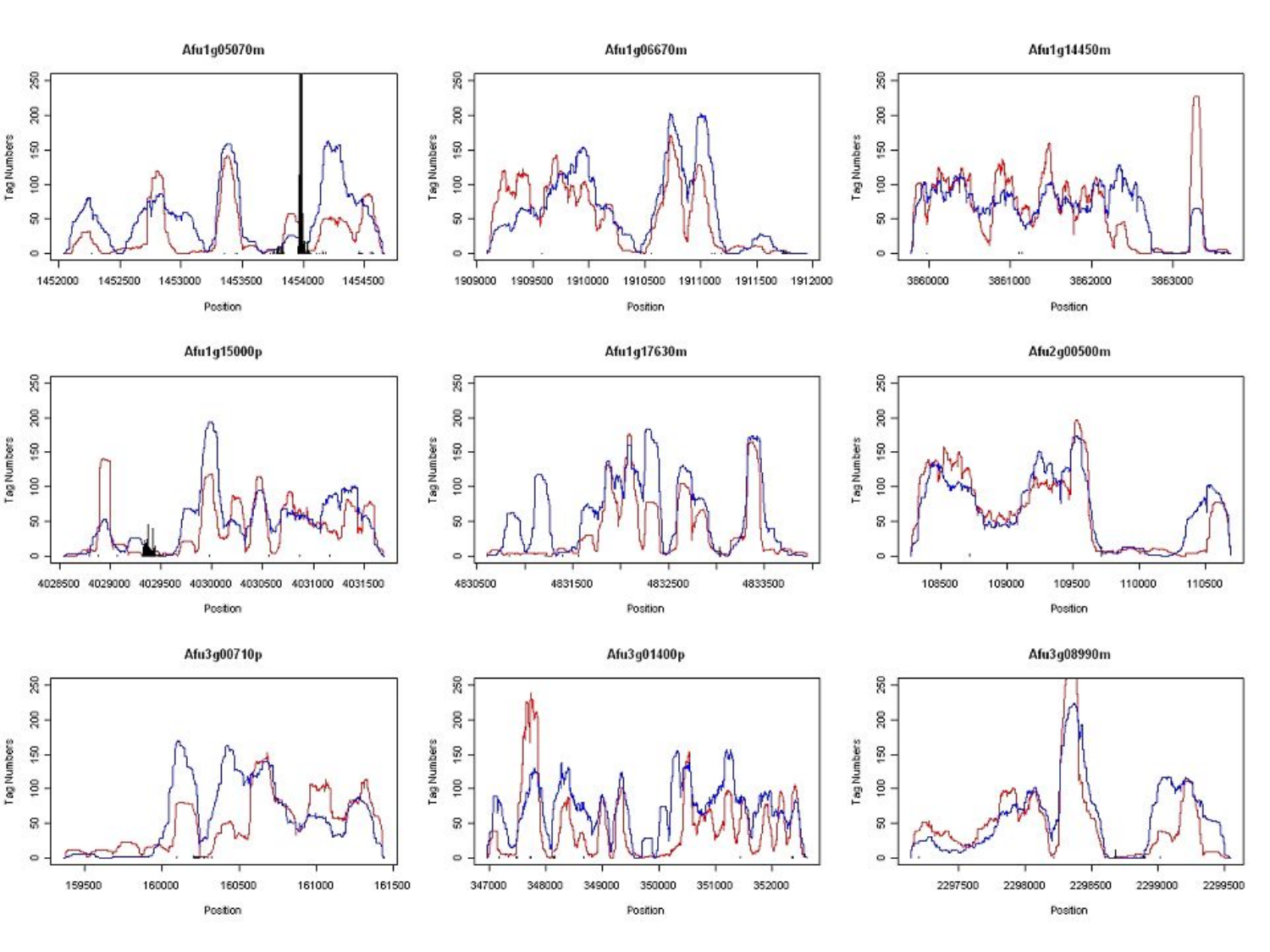

## Slide 2
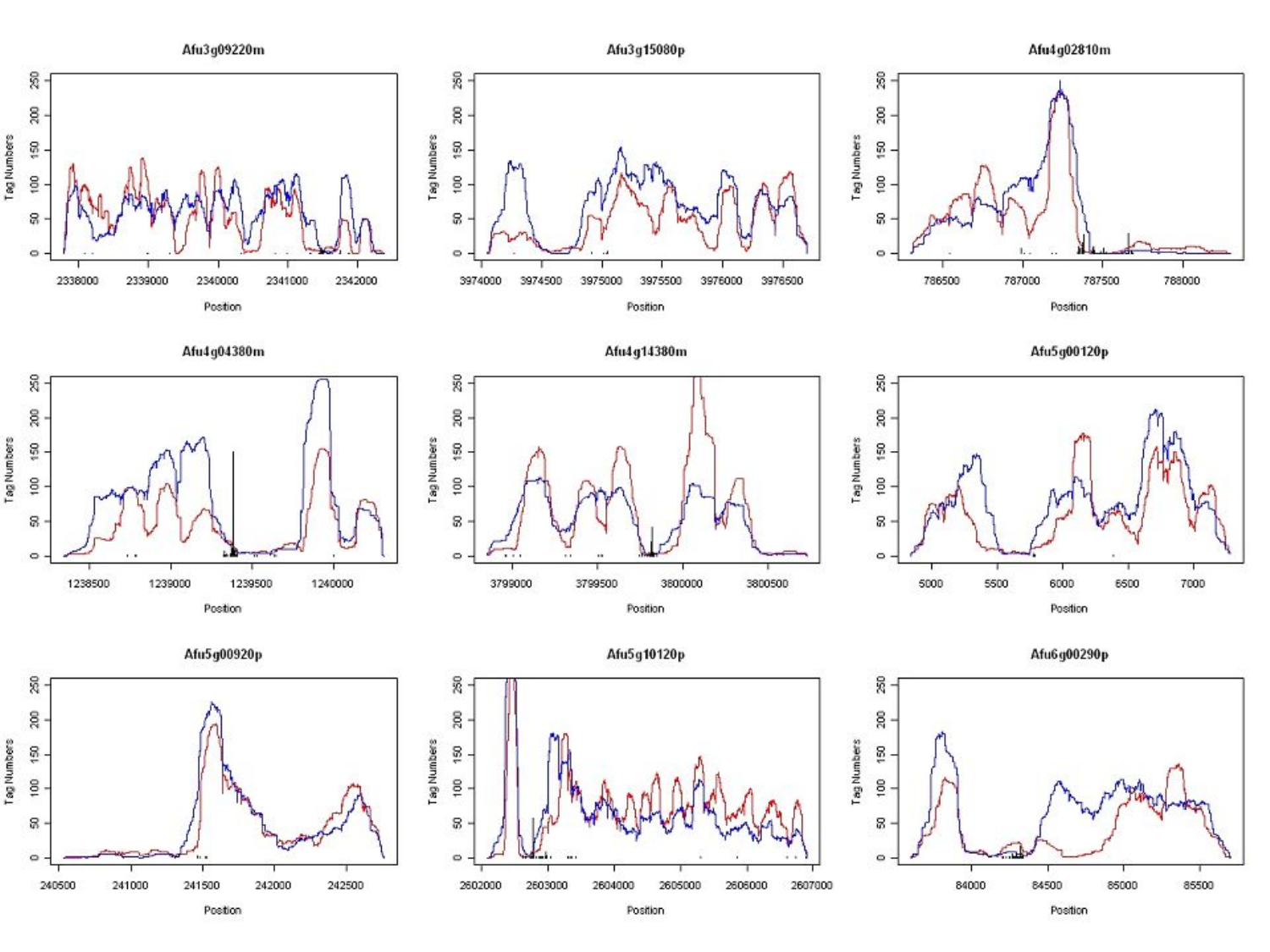

## Slide 3
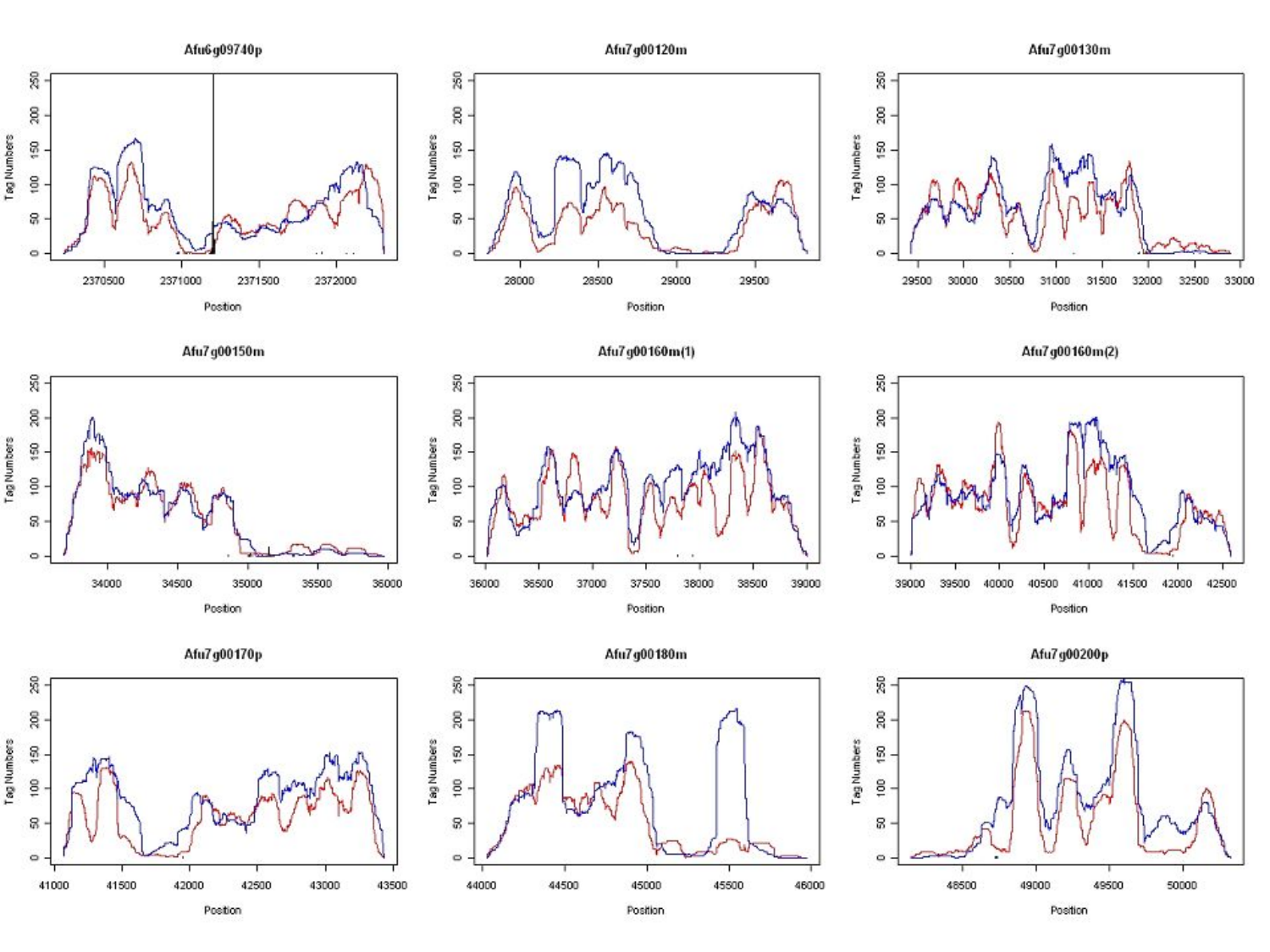

## Slide 4
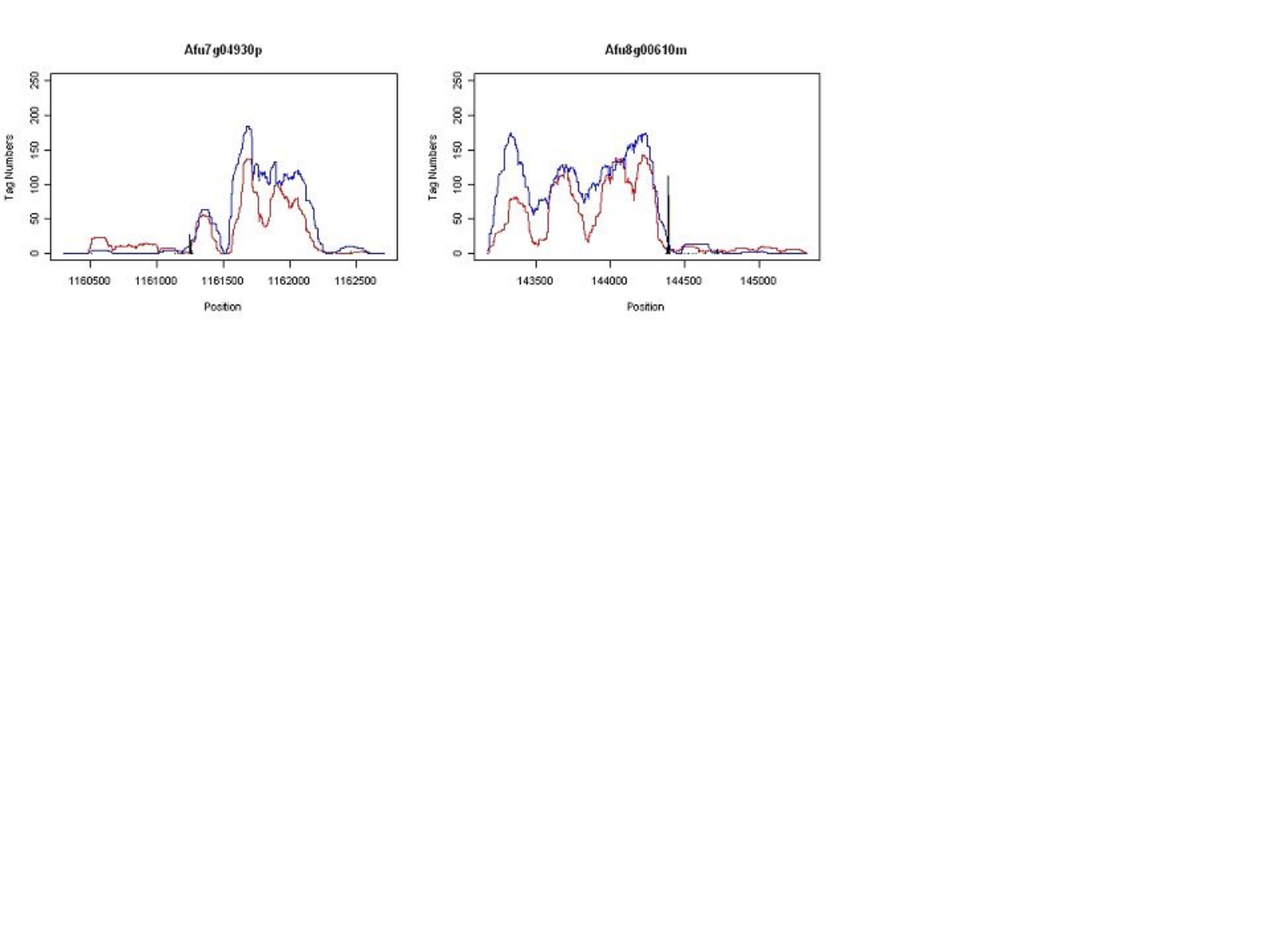

Supplement: Figure S4 — Mapping numbers of mononucleosomes and transcription start sites of the 28 up-regulated genes between the TSA-treated and untreated cells. Title indicates gene name with the last character “p” or “m”. The “p” indicates that the region between the positions 1 and 1,000 is the promoter and the other region is gene body. The “m” indicates that the region between the position 1,000 downstream from the last position and the last position is the promoter and the other region is gene body. Red and blue indicate the mononucleosome mapping number of the untreated cells and that of the TSA-treated cells respectively. The arrow indicates the region from the translational start to the end. The bars indicate the transcription start sites and the mapping numbers. (0.68 MB PPT) [file pone.0009916.s009.ppt]

## Slide 1
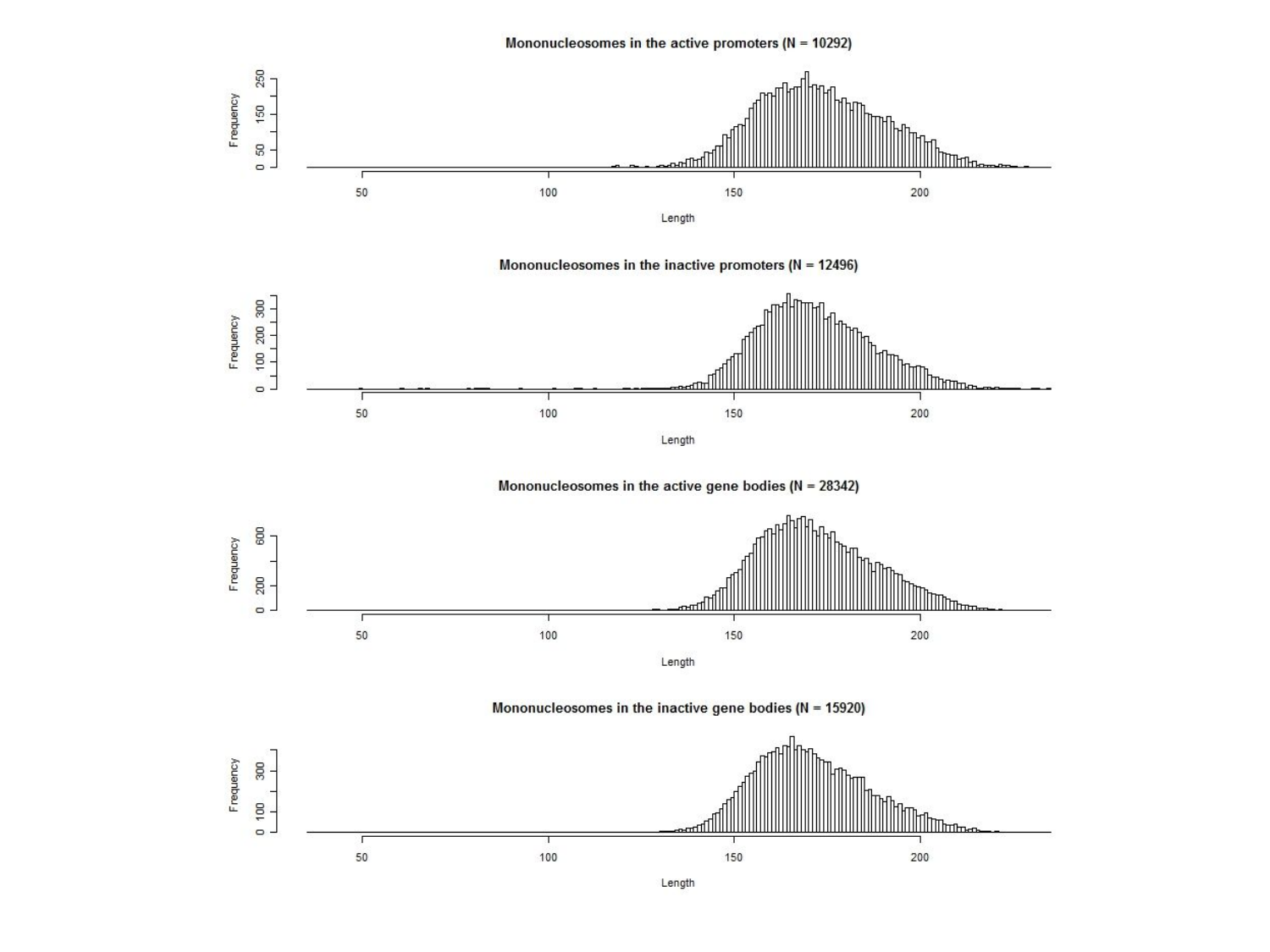

Supplement: Figure S5 — Histograms of mononucleosomal DNA fragment lengths in the promoters and bodies of transcriptionally active and inactive genes of the TSA-treated cells. We extracted highly expressed (active) genes (not including rRNA genes) and lowly expressed or silent (inactive) genes based on the microarray data of RNAs from the TSA-treated cells of Aspergillus fumigatus. (0.17 MB PPT) [file pone.0009916.s010.ppt]
